# *In silico* analysis reveals distinct changes in markers of epithelial to mesenchymal transition in glioma subtypes

**DOI:** 10.17305/bb.2025.12598

**Published:** 2025-07-17

**Authors:** Nives Pećina-Šlaus, Alja Zottel, Željko Škripek, Borna Puljko, Fran Dumančić, Anja Bukovac, Ivana Jovčevska, Anja Kafka

**Affiliations:** 1 Department of Biology, School of Medicine, University of Zagreb, Zagreb, Croatia; 2 Laboratory of Neuro-Oncology, Croatian Institute for Brain Research, School of Medicine, University of Zagreb, Zagreb, Croatia; 3 Center for Functional Genomics and Biochips, Institute of Biochemistry and Molecular Genetics, Faculty of Medicine, University of Ljubljana, Ljubljana, Slovenia; 4 Laboratory for Molecular Neurobiology and Neurochemistry, Croatian Institute for Brain Research, School of Medicine, University of Zagreb, Zagreb, Croatia; 5 Department of Chemistry and Biochemistry, School of Medicine, University of Zagreb, Zagreb, Croatia

**Keywords:** Glioma, EMT marker, *NOTCH1*, *SOX2*, progression, WHO grade, cBioPortal, GlioVis, quantitative real-time polymerase chain reaction, qRT-PCR.

## Abstract

Epithelial-to-mesenchymal transition (EMT) plays a critical role in tumor progression and metastasis, including in gliomas. To examine and interpret data on major genes involved in EMT and associate their changes with low-grade glioma (LGG) and/or high-grade glioma (HGG), data from the cBioPortal—a publicly available database for tumor genomics and transcriptomics, were collected for 13 genes: *CDH1, CDH2, CTNNB1, LEF1, NOTCH1, SNAI1, SNAI2, SOX2, TJP1/ZO1, TWIST1, VIM, ZEB1,* and *ZEB2*. The dataset included mutations, copy number alterations, and changes in transcript levels reported for each gene. The genes were additionally validated by gene expression on the GlioVis portal, STRING protein network analysis, survival analysis, and experimentally with quantitative real-time polymerase chain reaction (qRT-PCR). Glioblastoma (GBM) and diffuse glioma harbored changes in all 13 analyzed genes, while anaplastic oligodendroglioma and anaplastic astrocytoma in 46.15%, oligodendroglioma in 23.08%, and oligoastrocytoma in 15.38%. *NOTCH1* and *SOX2* were most affected by changes. The *NOTCH1* gene was statistically more frequently changed compared to *CDH1*, *CTNNB1,* and *ZEB1* (*P* < 0.05). The virtual study showed that alterations in *NOTCH1* and *LEF1* were associated with LGG, while alterations in *CDH1, CTNNB1, TJP1, TWIST1, SOX2*, *VIM, ZEB1,* and *ZEB2* were associated with HGG. Differential expression analysis stratified for *IDH1* mutations showed that *IDH1*-mutant GBM had significantly lower *CDH2, LEF1,* and *SNAI1* expression, and higher *ZEB1*. Gene expression in different GBM subtypes showed that the *TJP1/ZO1* gene was associated with the classical subtype, while *ZEB2* was associated with the proneural subtype. qRT-PCR confirmed GlioVis mRNA expression data for *NOTCH1, SOX2, CDH1, CTNNB1, TJP1/ZO-1, VIM, TWIST1,* and partially for *SNAI1 (SNAIL), SNAI2,* and *CDH2.* Our study shows consistent changes in genes involved in EMT in gliomas of different grades. Additional research is needed to confirm the knowledge brought by this study.

## Introduction

Epithelial-to-mesenchymal transition (EMT) is a critical molecular process through which cells acquire migratory capabilities, losing their epithelial characteristics and tissue integrity while adopting a mesenchymal phenotype [1, 2]. EMT plays a pivotal role in embryonic development, wound healing, tissue fibrosis, and tumorigenesis. In the context of tumorigenesis, EMT contributes to the invasiveness and metastatic potential of tumors [3]. However, the conventional binary classification of cells into epithelial and mesenchymal phenotypes inadequately captures the complexity of EMT observed in clinical scenarios. This complexity is underscored by various distinct molecular processes involved, prompting a re-evaluation of EMT as a “spectrum” [4, 5]. Recent studies have identified a hybrid (partial) EMT state characterized by the presence of both mesenchymal and epithelial features, which is associated with increased cellular plasticity, collective migration, stemness properties, and enhanced metastatic potential [6–8]. To elucidate the roles of epithelial and mesenchymal markers, as well as the activation of various EMT-related transcription factors (EMT-TFs), we focused on the following genes, all of which are crucial in the EMT process: *CDH1, CDH2, TJP1/ZO-1, CTNNB1, LEF1, NOTCH1, SNAI1, SNAI2, SOX2, TWIST1, VIM, ZEB1*, and *ZEB2*. Key markers of the epithelial phenotype include E-cadherin, encoded by the *CDH1* gene, and tight junction protein-1, encoded by the *TJP1/ZO-1* gene (TJP1, *Zonula occludens-1*, ZO-1). The primary marker of the mesenchymal phenotype is N-cadherin, encoded by the *CDH2* gene [9], along with vimentin (encoded by *VIM*), beta-catenin (gene *CTNNB1*), Lymphoid Enhancer Binding Factor 1 (*LEF1*), and *NOTCH1*. Additional genes encoding EMT-TFs were also included in the study, *SOX2, TWIST1, SNAI1, SNAI2, ZEB1,* and *ZEB2*. EMT also plays a role in glioma tumors.

Gliomas are one of the most common intracranial tumors with great aggressiveness and invasiveness. A major factor contributing to the high invasiveness of glioma cells is their acquisition of mesenchymal traits, which facilitate invasion and migration [1, 10]. Gliomas, which are primary tumors of the central nervous system (CNS), originate from glial cells. All gliomas are classified into a single category based on mitotic activity, diffuse growth patterns, and the mutational status of the *IDH1* and *IDH2* genes, along with several other molecular biomarker assessments. According to the World Health Organization (WHO) [11–13], tumors are categorized into four grades. Tumors are graded within tumor types rather than across different types [13]. The prognosis for diffuse gliomas is influenced by various factors, including tumor grade. Grades documented on cBioPortal and included in our analysis are: diffuse gliomas (grade 2), oligodendrogliomas (grade 2), oligoastrocytomas (grade 2), anaplastic oligodendroglioma (grade 3), anaplastic astrocytoma (grade 3), and glioblastoma (GBM) (grade 4). Gliomas can also be stratified into low-grade gliomas (LGGs, encompassing grades 1 and 2) and high-grade gliomas (HGGs, encompassing grades 3 and 4). LGGs predominantly affect young adults, grow more slowly, and are associated with a more favorable prognosis compared to HGGs.

Data from multiple studies available in the cBioPortal public database were analyzed *in silico*. We compared molecular markers of EMT between the LGG and HGG groups, hypothesizing that specific alterations in genes encoding mesenchymal phenotype markers correlate with higher glioma grades, while changes in epithelial marker genes are associated with lower grades. We collected data on mutations, amplifications, and deletions, analyzing the specific types and frequencies of changes for each selected gene. The observed changes reported in cBioPortal were validated through additional database searches and quantitative real-time polymerase chain reaction (qRT-PCR). We also performed *in silico* analyses of gene expression across different glioma grades, survival analyses, and protein network analyses.

## Materials and methods

### cBioPortal

The analysis of selected genes in gliomas of various pathohistological types and grades was conducted using data from The cBioPortal for Cancer Genomics database [14, 15]. This analysis encompassed stored data on mutations, copy number alterations (CNAs), and mRNA expression.

### Analyzed studies

Eight studies from the cBioPortal database were included, comprising a total of 3497 samples obtained from 3143 patients. Collectively, these studies identified gene alterations in 379 (12%) of the queried patients and 395 (11%) of the queried samples. The selected studies included: *Diffuse Glioma* (GLASS Consortium, Nature 2019) [16]—whole genome or whole exome sequencing analysis of 444 adult patients; *Glioma* (MSK, Clin Cancer Res 2019) [17]—targeted sequencing on MSK-IMPACT and FMI Panels of 1004 samples; *LGGs* (UCSF, Science 2014) [18]—whole exome sequencing of 61 samples; *Merged Cohort of LGG and GBM* (The Cancer Genome Atlas (TCGA), Cell 2016) [19]—whole exome sequencing of 1122 LGG and GBM tumor/normal pairs; *Brain Tumor Patient-Derived Xenografts* (PDXs) (Mayo Clinic, Clin Cancer Res 2020) [20]—whole exome sequencing of 106 samples; *GBM* (CPTAC, Cell 2021) [21]—proteogenomic and metabolomic characterization of human GBM, including whole genome or whole exome sequencing of 99 samples generated by CPTAC; *GBM* (Columbia, Nat Med. 2019) [22]—whole-exome sequencing of 42 GBM samples with matched normals; and *GBM Multiforme* (TCGA, Firehose Legacy)—619 samples sourced from GDAC Firehose, previously known as TCGA Provisional [14, 15]. Data on mutations, CNAs, and mRNA transcript levels for each gene were downloaded and examined as part of a comprehensive study. All cBioPortal data adhered to uniform clinical criteria and underwent consistent processing and normalization, facilitating comparative analysis across different studies. Following the creation of a virtual study, graphical representations of gene analyses were generated using Excel 2016 (Microsoft). Our virtual study, completed in March 2025, provides downloadable data available at [23]. The current reference version of the human genome utilized by cBioPortal is hg19/GRCh37. RNA and DNA data were procured from tumor samples and adjacent normal tissue using an adapted DNA/RNA AllPrep kit (QIAGEN). Pathologists systematically reviewed the specimens to confirm histopathological diagnoses according to the latest edition of the WHO classification for each tumor type. Copy number data were generated using Affymetrix SNP 6.0 arrays following standard protocols from the Broad Institute Genome Analysis Platform. CNAs represent continuous gene copy number values calculated as the difference between the copy number of the tumor gene and the reference. Normalized continuous CNA values were processed using the copy-number analysis algorithm *Genomic Identification of Significant Targets in Cancer* (GISTIC 2.0), indicating the copy-number level per gene. Continuous values of −2 were classified as deep deletions (indicating a homozygous deletion), while values of −1 represented shallow deletions (indicating a heterozygous deletion). Samples with a continuous CNA value of 0 were designated as diploid, with no gene copy number changes. A value of 1 indicated a low-level gain (a few additional copies, often broad), and amplifications were characterized by a value of 2, indicating high-level amplification (more copies, often focal).

The cBioPortal ensures comparability across datasets. Data from the PanCancer Atlas are categorized by tumor type, but these studies share uniform clinical elements, consistent processing, and normalization of mutations, CNAs, and mRNA data, thereby facilitating comparative analyses.

All samples were statistically processed according to the following variables: pathohistological diagnosis, frequency, type of changes (mutation, CNA), and malignancy grade. Statistical analyses were performed using IBM SPSS Statistics 23.0 software (SPSS, Chicago, IL, USA), with significance set at *P* < 0.05. Gene alterations were analyzed in specific tumor types using Fisher’s exact test. Correction for multiple comparisons was conducted using the Benjamini–Hochberg false discovery rate (FDR) method.

### Gene expression in different glioma grades

Gene expression analysis across various glioma grades (WHO grades 2–4) was performed using the GlioVis online tool, which includes the TCGA_GBMLGG dataset (https://gliovis.bioinfo.cnio.es/). To assess expression differences among different glioma types and GBM subtypes, a one-way analysis of variance (ANOVA) was conducted independently for each gene. A Tukey’s honest significant difference (HSD) test was employed for *post hoc* comparisons between groups [24]. The strength of subtype effects was quantified using η^2^ (eta squared) effect sizes calculated from the ANOVA models. To correct for multiple testing across all genes, ANOVA *P* values were adjusted using the Benjamini–Hochberg FDR method, which was also applied to all pairwise comparisons from the Tukey HSD tests.

### Gene expression in GBM

The *GBM Multiforme* study (TCGA, Firehose Legacy) was utilized for mRNA expression analysis, which included data for 619 samples.

For mRNA expression level data, next-generation RNASeq V2 RSEM (*RNA-seq by Expectation Maximization*) sequencing was downloaded [25]. RNASeq V2 from TCGA is processed and normalized using the software RSEM. Specifically, the RNASeq V2 data in cBioPortal corresponds to the rsem.genes.normalized_results file from TCGA. A more detailed explanation of RSEM output can be found at https://www.biostars.org/p/106127/. cBioPortal then calculates *z*-scores. The expression data assigned from Illumina were batch-corrected to adjust for platform variations between the GAII and HiSeq Illumina sequencers. Additional corrections were made for various sequencing centers [26]. More precisely, the RNASeq V2 data in cBioPortal matches *the rsem.genes.normalized_results* file from TCGA. cBioPortal mRNA expression data are calculated as the relative expression of a specific gene in a tumor sample to the gene’s expression distribution in a reference (all samples that are diploid for the gene in question) population of samples [15].

During the data normalization process, expression data (RPPA) for proteins were batch effects-corrected and median-centered in both directions. Within cBioPortal, the protein data were additionally processed and normalized with the calculation of the *z*-scores and converted to the log scale.

### Differential analyses stratified according to *IDH1* status and glioma subtypes

Differential expression analysis was performed on the TCGA_GBMLGG dataset (obtained from Gliovis [24]), stratified by grade 2, grade 3, GBM *IDH1* wt (wild type), and GBM *IDH1* mut (mutated).

Subtype analysis was also conducted on the GlioVis TCGA_GBMLGG dataset, filtered for GBM *IDH1* wt only. Both analyses were performed using R (4.4.0) and RStudio (2023.06.0). Statistical significance was determined by the one-way ANOVA statistical test.

### Validation by qRT-PCR

Glioma samples graded from 2–4 were collected from the University Hospital Center “Zagreb,” University Hospital Center “Sestre Milosrdnice,” Zagreb, and University Medical Centre Ljubljana. Certified neuropathologists set the accurate diagnosis in concordance with the most recent WHO classification [12]. The patients included in the study had no family history of brain tumors and did not undergo any cancer treatment prior to surgery that could affect the results of qRT-PCR analyses. Altogether, there were 18 samples grade 2 (LGG), of which 17 were *IDH1* mutant and one was wild type (mean age ═ 40.22). HGG gliomas consisted of five samples grade 3 of which four were *IDH1* mutant and one was wild type. Sixteen samples were grade 4 (GBM), of which two were *IDH1* mutant and 12 were wild type (two samples were not determined for *IDH1*). The mean age of HGG patients was 55.7 years. Furthermore, 12 non-tumor reference brain tissues were collected as qRT-PCR controls.

qRT-PCR was performed to validate the candidate genes. Experimental validation by qRT-PCR was performed on normal brain tissues, LGG and HGG tissue samples. The GeneJET RNA Purification kit (Thermo Fisher Scientific #K0702) was used to extract total RNA from brain tissue samples from both healthy and tumorous subjects, while some of the RNA was already isolated as described in [27]. The High-Capacity cDNA Reverse Transcription Kit with RNase Inhibitor (Applied Biosystems #4388950) was used to reverse-transcribe equal amounts of RNA after it had been treated with DNase I (Sigma-Aldrich #EN0521). Each sample was subjected to qRT-PCR analysis using a constant quantity of cDNA with the qRT-PCR SYBR Green PCR (Applied Biosystems, #4309155) or TaqMan Fast Advanced Mastermix (Thermofisher, #4444557)-based qRT-PCR. Table S1 lists the primer sequences that were employed. The relative quantification approach (ΔΔCt) was utilized to determine the target gene expression for group comparisons, normalized per beta-actin as an endogenous control. To determine the target gene expression for group comparisons, a 7900 HT Real-Time PCR System (Applied Biosystems) or QuantStudio 7 Pro (ThermoFisher) was utilized for real-time fluorescence detection.

#### qRT-PCR validation of *SOX2* and *NOTCH1*

For *SOX2* and *NOTCH1* validation, RNA had been previously extracted as described in earlier studies [27]. For each sample, 500 ng of total RNA was treated with DNase I (Roche) at 30 ^∘^C for 15 min, followed by enzyme inactivation at 75 ^∘^C for 10 min. cDNA was synthesized using the High-Capacity cDNA Reverse Transcription Kit (Thermo Fisher Scientific), with the addition of RNase inhibitor (1 µL per reaction; Cat. No. N8080119, Thermo Fisher). The reverse transcription protocol was carried out under the following conditions: 10 min at 25 ^∘^C, 120 min at 37 ^∘^C, and 5 min at 85 ^∘^C. qRT-PCR was performed using TaqMan assays in a 5 µL reaction volume, containing 0.25 µL of the TaqMan gene expression assay, 2.5 µL of TaqMan Gene Expression Master Mix, 2 µL of nuclease-free water, and 0.25 µL of diluted cDNA. Thermal cycling was carried out under the following conditions: 95 ^∘^C for 20 s for initial denaturation, followed by 45 cycles of 95 ^∘^C for 1 s and 60 ^∘^C for 20 s, with a final hold at 4 ^∘^C. All reactions were run in technical triplicates. The probes used in the study were as follows: GAPDH Hs99999905_m1, HPRT1Hs02800695_m1, SOX2 Hs04234836_s1, and NOTCH1 Hs01062014_m1 (all from ThermoFisher). Data were analyzed according to MIQE guidelines [28]. qRT-PCR data are shown as X ± SEM. Data were analyzed as described before [29]. First, data was checked for normality using the Shapiro–Wilk test. As the data did not follow a normal distribution, a non-parametric Kruskal–Wallis test with Dunn’s multiple comparisons test was applied. A significance threshold was set at 0.05. GraphPad Prism version 9 was used to process and display all of the data graphically (GraphPad Software Inc.).

### Survival analyses

We performed age-adjusted survival analysis for samples that were *IDH1* wild type (wt) and those that carried *IDH1* mutations. Survival analysis was conducted on the TCGA_GBMLGG dataset obtained from GlioVis [24], separately for GBM *IDH1* wild-type and GBM *IDH1* mutant samples. Patients were stratified into high and low expression groups for each gene based on median expression. Kaplan–Meier curves were generated and compared using the log-rank test. Multivariate Cox proportional hazards models were used to estimate hazard ratios (HRs) and 95% confidence intervals (CIs), adjusting for age. All analyses were performed in R (v4.4.0) using RStudio (2023.06.0) with the survival and survminer packages.

### Protein network analysis

Protein network analysis was performed using the STRING online tool [30]. The following genes were included in the analysis: *CDH1, CDH2, TJP1/ZO-1, CTNNB1, LEF1, NOTCH1, SNAI1, SNAI2, SOX2, TWIST1, VIM, ZEB1, ZEB2*. The interaction score was set to 0.9 (highest confidence) [31].

### Ethical statement

The Ethics Committees of the School of Medicine, University of Zagreb (Case number: 380-59-10106-20-111/126; Class: 641-01/20-02/01), University Hospital Center Zagreb (Case number: 02/21 AG; Class: 8.1-20/108-2), and University Hospital Center “Sestre Milosrdnice” (Case number: 251-29-11-20-01-9; Class: 003-06/20-03/015) have approved the research. The use of human tissue samples was approved by the National Medical Ethics Committee of the Republic of Slovenia (Approval Numbers: 92/06/12, 89/04/13, and 95/09/15). Reference samples were collected during autopsies in accordance with the legal regulations of the Republic of Slovenia. All samples used in this study are anonymized. The study adhered to the principles outlined in the Declaration of Helsinki, with patients consenting to participate.

Data retrieved from the publicly available cBioPortal database do not require ethical approval. All patients whose samples were used in this analysis signed informed consent. Since the data are not identifiable, secondary data analysis does not require additional ethical approval, as it was already obtained in original analyses [16–22]. The secondary data analysis was performed in compliance with the World Medical Association Declaration of Helsinki on Ethical Principles for Medical Research Involving Human Subjects. The data is properly anonymized, making it impossible to identify individuals.

### Pathway enrichment analysis

To explore how these genes interact within EMT pathways, enrichment analysis was performed to indicate pathways in which the selected EMT genes are involved. The genes were analyzed with the NDEx online tool (https://cytoscape.org/).

## Results

### The overview of genetic changes distributed to glioma type

Our first analysis summarized all genetic changes reported for gliomas. Table 1 provides an overview of a pooled analysis that eight studies found in 13 genes involved in EMT in LGG and HGG. Mutations prevailed over amplifications and deep deletions, while a small number of gliomas harbored multiple changes. Anaplastic oligodendrogliomas (grade 3) contained the highest percentage of mutations, 29.03% (18/62 cases), and oligodendrogliomas followed with 16.79%. Anaplastic astrocytomas harbored 7.58%, oligoastrocytomas 6.25%, while mutations in GBM were present in 5.91% (98/1659 cases) and diffuse glioma in 4.91%. Although the percentage of amplifications for oligoastrocytomas was high at 6.25% (1/16 cases), this frequency should be taken with caution since only 16 samples were available. However, when diffuse gliomas, another subtype of grade 2 glioma is observed, amplifications were found in 4.91% (65/1324) showing that amplifications were associated with grade 2 gliomas. Deep deletions were found in 0.54% of GBMs and in 0.98% of diffuse gliomas. Multiple changes were reported in 0.23% of diffuse gliomas and 0.12% of GBM cases.

**Table 1:** Summary results of the analysis of all genes

	**Glioma type**					
	**Anaplastic oligodendro-** **glioma (grade 3)**	**Oligodendroglioma (grade 2)**	**Anaplastic astrocytoma (grade 3)**	**Glioblastoma (grade 4)**	**Oligoastrocytoma (grade 2)**	**Diffuse glioma (grade 2)**
*Change*						
Mutations	29.03%	16.79%	7.58%	5.91%	6.25%	4.91%
Amplifications	1.61%	2.92%	4.04%	4.00%	6.25%	4.91%
Deep deletions				0.54%		0.98%
Multiple alterations				0.12%		0.23%
Total		19.71%	11.62%	10.5%	12.5%	11.03%

### Changes in the *CDH1* and *CDH2* genes

**Figure 1.**
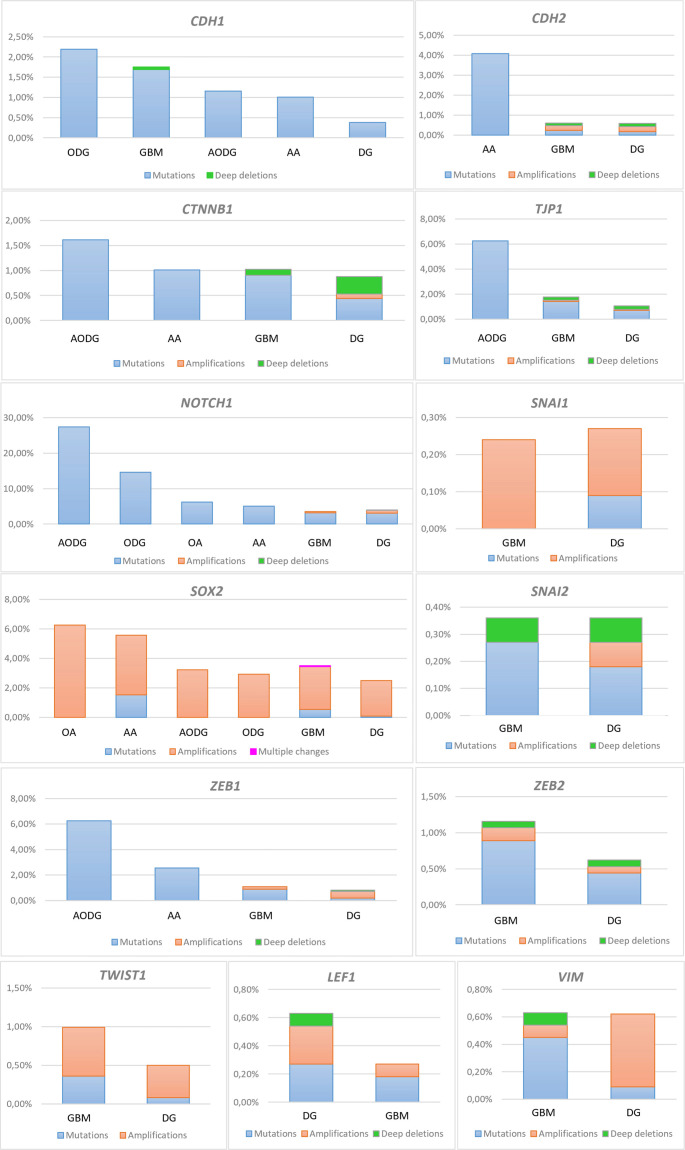
**Results of gene analyses:** ***CDH1; CDH2; TJP1/ZO-1; CTNNB1; LEF1; VIM; NOTCH1; SNAI1; SNAI2; TWIST1; SOX2; ZEB1; ZEB2***. The *Y*-axis denotes the frequency of observed changes, and the *X*-axis glioma type. DG: Diffuse glioma; ODG: Oligodendroglioma; OA: Oligoastrocytoma; AODG: Anaplastic oligodendroglioma; AA: Anaplastic astrocytoma; GBM: Glioblastoma; LEF1: Lymphoid Enhancer Binding Factor 1.

Mutations were a predominant type of change for the *CDH1* gene, and only one case of GBM (0.06%) harbored a deep deletion. *CDH1* was most often mutated in oligodendrogliomas, 2.19% (3/137 cases), and GBMs followed with 1.69% (28 cases). As the grade of glioma decreased, so did the number of cases in which *CDH1* was mutated; thus, anaplastic oligodendrogliomas harbored 1.16% and anaplastic astrocytomas 1.01%. The lowest number of mutations was present in diffuse gliomas (grade 2) with only 0.38% (5/1324 cases) (Figure 1). Seven mutations were characterized as drivers and oncogenic.

The results of the *CDH2* gene analysis were somewhat reciprocal to those obtained for the *CDH1* gene (Figure 1). For instance, *CDH1* mutations predominated in oligodendroglioma and GBM, while *CDH2* mutations were not reported for oligodendroglioma. Mutations were present in only 0.24% and 0.17% of GBM and diffuse glioma, respectively. *CDH2* was mutated most frequently (4.08%) in anaplastic astrocytoma (grade 3). Mutations were of unknown significance, and none were characterized as oncogenic. Contrary to *CDH1*, where no amplifications were found, amplifications of *CDH2* were present in a small percentage of diffuse gliomas and GBM, at 0.25% and 0.24%, respectively. Deep deletions of *CDH2* were also recorded in these two types: in diffuse glioma at 0.17%, and GBM at 0.12% (Figure 1).

### *TJP1/ZO-1* gene changes

The gene for the tight junction adapter protein, *TJP1/ZO-1*, was mutated, amplified, and deeply deleted. Again, mutations were the most common change. Thirty different mutations were all of unknown significance. They were most pronounced in anaplastic oligodendroglioma (grade 3) at 6.25%. Altogether, the *TJP1/ZO-1* gene was mutated in 1.43% (16 cases), amplified in 1 case (0.09%), and deeply deleted in 0.27% (3 cases) of GBMs (Figure 1). Diffuse gliomas harbored 0.71% of mutations, 0.09% of amplifications, and 0.27% of deep deletions (Figure 1).

### *CTNNB1* gene changes

Predominant alterations of *CTNNB1* were mutations. Of the 26 mutations reported, one was an oncogenic driver, and the rest were of unknown significance. They were found in 1.61% of anaplastic oligodendroglioma, 1.01% of anaplastic astrocytoma, 0.90% of GBM, and 0.44% of diffuse glioma. The results obtained for the *CTNNB1* gene show that the changes were most frequently confined to grade 3 gliomas (Figure 1). Deep deletions were reported for GBM and diffuse glioma at 0.12% and 0.35%, respectively. Only one diffuse glioma (grade 2) (0.09%) harbored amplification of *CTNNB1* (Figure 1).

### *LEF1* gene changes

The changes in the *LEF1* gene were confined to diffuse glioma and GBM. Both mutations and amplifications were more frequent in diffuse glioma compared to GBM. Mutations of unknown significance were present in 0.27% of diffuse glioma vs 0.18% in GBM. Amplifications in diffuse glioma (grade 2) amounted to 0.27%, compared to 0.09% found in GBM. There was also one diffuse glioma (0.09%) with a deep deletion of this gene (Figure 1).

### *VIM* gene changes

The changes in the *VIM* gene (vimentin), including mutations, amplifications, and deep deletions, were differently distributed in patients with GBM compared to diffuse gliomas. In GBM, the gene was mutated in 0.45% (5/1120 cases), amplified in 0.09% (1 case), and deleted in one case (0.09%). On the other hand, in diffuse gliomas (grade 2) it was amplified (0.53%; 6/1131 cases) more often than mutated (0.09%). The mutations were of unknown significance (Figure 1).

### *NOTCH1* gene changes

The *NOTCH1* gene showed a high percent of changes across all types of gliomas. The obtained results indicated that the prevalent type of changes were mutations, while amplifications and deep deletions were extremely rare. There were 193 mutations, of which 72 were characterized as oncogenic drivers. The largest number of mutations was registered in anaplastic oligodendroglioma (grade 3) at 27.42% (17 cases), followed by mutations in oligodendroglioma (14.60%), oligoastrocytoma (6.25%), anaplastic astrocytoma (5.05%), and diffuse gliomas (3.1%). GBMs harbored mutations in 3.13% (52/1659 cases), while amplifications were present in 0.42% (7/1659 cases). Diffuse gliomas also harbored amplifications in 0.76% and deep deletions in 0.15% (Figure 1).

### *SNAI1* and *SNAI2* gene changes

Interesting results were obtained for the *SNAI1* gene, which was changed only in diffuse glioma and GBM. Namely, unlike previous genes where mutations prevailed, here amplifications were the most common changes. They were more frequent in GBM compared to diffuse gliomas. *SNAI1* gene amplification in GBMs occurs in 0.24% (2 cases), and in diffuse gliomas in 0.18% (2 cases). Mutations in diffuse glioma were recorded in only 0.09% and were not characterized as oncogenic (Figure 1). The next gene included in the analysis is the transcriptional repressor *SNAI2*. Similar to *LEF1* and *SNAI1*, changes in this gene have been reported only in GBM and diffuse gliomas. However, unlike *SNAI1*, where amplifications predominated, here mutations were most common. The gene was more often mutated in GBM compared to diffuse gliomas, at 0.27% (3/1120 cases) vs 0.18% (2/1131 cases). Only 0.09% of diffuse gliomas showed amplification. In contrast to *SNAI1,* there is the presence of deep deletions of *SNAI2* in both GBM (0.09%) and diffuse glioma (0.09%) (Figure 1).

### *TWIST1* gene changes

*TWIST1* changes were more frequent in GBM compared to diffuse glioma. Amplifications prevailed over mutations. In diffuse gliomas (grade 2), the gene was amplified in 0.42% and mutated in 0.08%. In GBM, the gene was amplified in 0.63% and mutated in 0.36% of samples (Figure 1).

### *SOX2* gene changes

The results of the analysis of the *SOX2* gene were similar to those of the *SNAI1* gene, with respect that both genes were most frequently amplified. The highest number of amplifications was found in 6.25% of oligoastrocytomas (grade 2) and 4.04% (eight cases) of anaplastic astrocytomas (grade 3). Anaplastic oligodendrogliomas harbored amplifications in 3.23%, and oligodendrogliomas in 2.92%, while in GBM they were present in 2.9% of cases and diffuse glioma in 2.42%. *SOX2* mutations were present in 1.52% of anaplastic astrocytomas, 0.54% of GBMs, and 0.076% of diffuse gliomas. One case (0.06%) of GBM showed multiple *SOX2* gene changes (Figure 1). Missense mutations of unknown significance predominated.

### *ZEB1* and *ZEB2* gene changes

The highest percentage of *ZEB1* mutations (6.25%) was associated with anaplastic oligodendrogliomas (grade 3). Anaplastic astrocytomas (grade 3) followed with 2.56%, and GBM with 0.89%, while diffuse gliomas had 0.18%. All of the mutations are of unknown significance. In addition to mutations, GBMs and diffuse gliomas also harbored amplifications (0.18% and 0.53%). Deep deletion of the *ZEB1* gene was present in one diffuse glioma (0.09%) (Figure 1).

The last gene included in the analysis was *ZEB2*. Unlike *ZEB1*, where GBM ranked third in terms of the frequency of gene changes, here it ranked first. Mutations prevailed in GBMs at 0.89%, while amplifications were present in 0.18%, and deep deletions in 0.09%. Changes of *ZEB2* in diffuse gliomas (grade 2) were less frequent. The gene was mutated in 0.44%, amplified in one case (0.09%), and deeply deleted in one case (Figure 1).

### Collective results of genetic changes distributed to LGG and HGG

**Figure 2.**
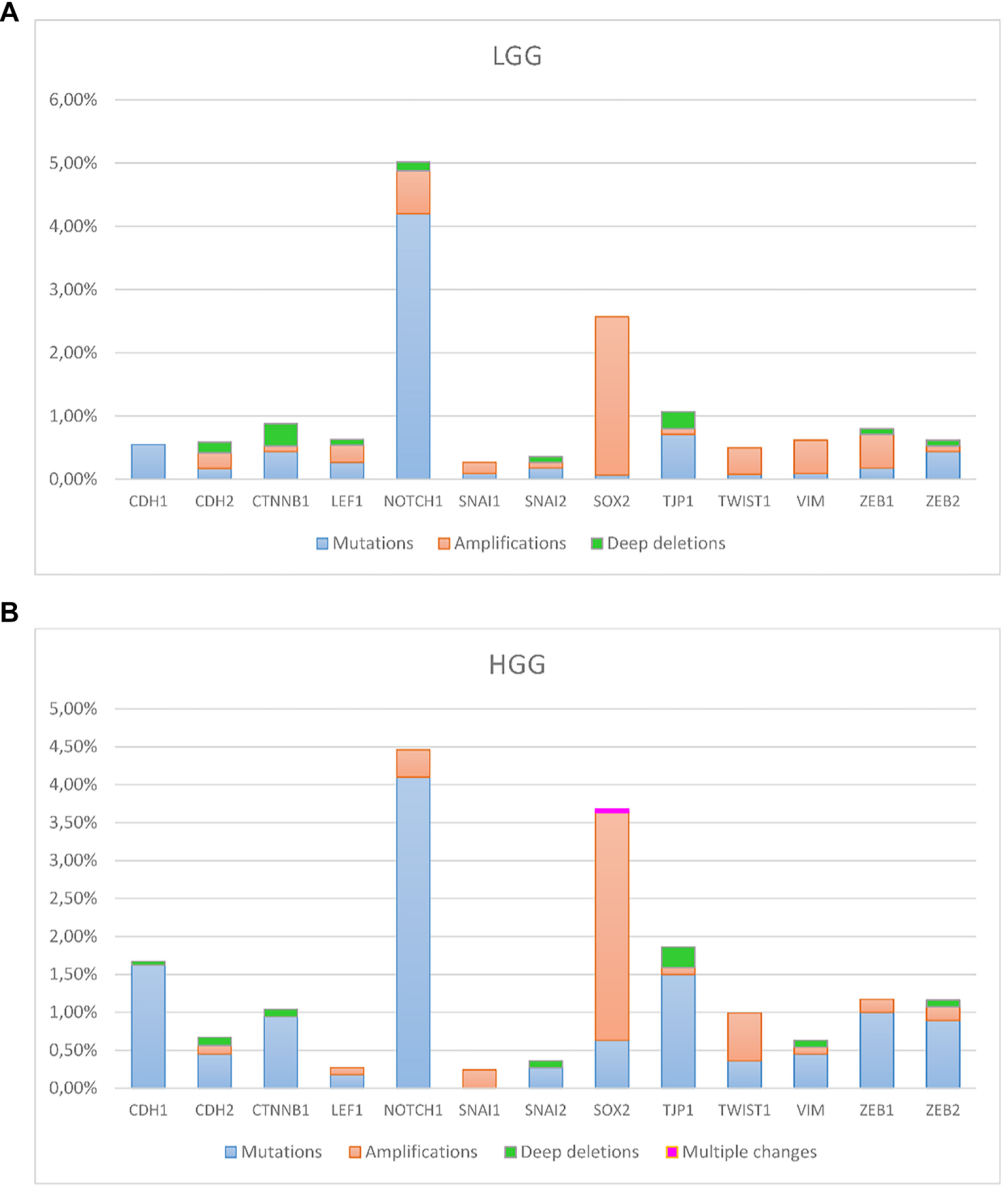
**The overall presentation of changes in LGG (A) and HGG (B).** The *Y*-axis denotes the frequency of observed changes, and the *X*-axis represents the genes. The type of changes is color-coded in the legend. LGG: Low-grade glioma; HGG: High-grade glioma.

To illustrate all the changes associated with each gene and divide them into LGG and HGG groups, we made a summary in Figure 2. From the figure, it is evident that in both groups of gliomas, the *NOTCH1* and *SOX2* genes were most affected by changes. *CDH1, CTNNB1, TJP1/ZO-1, ZEB1,* and *ZEB2* mutations were more common in HGGs. Only GBM and diffuse glioma had changes in all 13 analyzed genes. Anaplastic oligodendroglioma and anaplastic astrocytoma harbored changes in 6/13 (46.15%), oligodendroglioma in 3/13 (23.08%), and oligoastrocytoma in 2/13 (15.38 %) of the analyzed genes. In less than half (6/13 or 46%) of the analyzed genes, changes were distributed only in GBM and diffuse glioma. In seven analyzed genes: *CDH1, CDH2, CTNNB1, NOTCH1, SOX2, TJP1/ZO-1,* and *ZEB1*, changes were present in several pathohistological diagnoses ranging from 3 to all 6. Genes in which changes were present in all six pathohistological diagnoses were *NOTCH1* and *SOX2*. Changes of the *CDH1* gene were present in 5/6 glioma types, of *CTNNB1* and *ZEB1* genes in 4/6 pathohistological types, and of *CDH2* and *TJP1/ZO-1*, in 3/6 glioma types. Changes in *NOTCH1* and *SOX2* were present in all glioma types. Furthermore, the frequency of changes in those genes where changes were present was statistically significantly higher in the *NOTCH1* gene than in the *CDH1* gene in anaplastic oligodendroglioma (Benjamini–Hochberg Adjusted *P* value, significant using an FDR of 0.05 (*P* < 0.05), oligodendroglioma (*P* < 0.05), GBM (*P* < 0.05), anaplastic astrocytoma (*P* < 0.05), and diffuse glioma (*P* < 0.05). The same trend was present when the frequency of changes in the *NOTCH1* gene was compared with that of the *CTNNB1* gene—the frequency of changes in the *NOTCH1* gene was significantly higher in anaplastic oligodendroglioma (*P* < 0.05), GBM (*P* < 0.05), anaplastic astrocytoma (*P* < 0.05), and diffuse glioma (*P* < 0.05). A comparison of the frequency of changes in the *NOTCH1* gene and the *ZEB1* gene shows that the frequency of changes in the *NOTCH1* gene was significantly higher in GBM (*P* < 0.05) and diffuse glioma (*P* < 0.05). However, in anaplastic oligodendroglioma and anaplastic astrocytoma, the frequency of changes in these two genes was similar.

### Gene expression in different glioma grades

**Figure 3.**
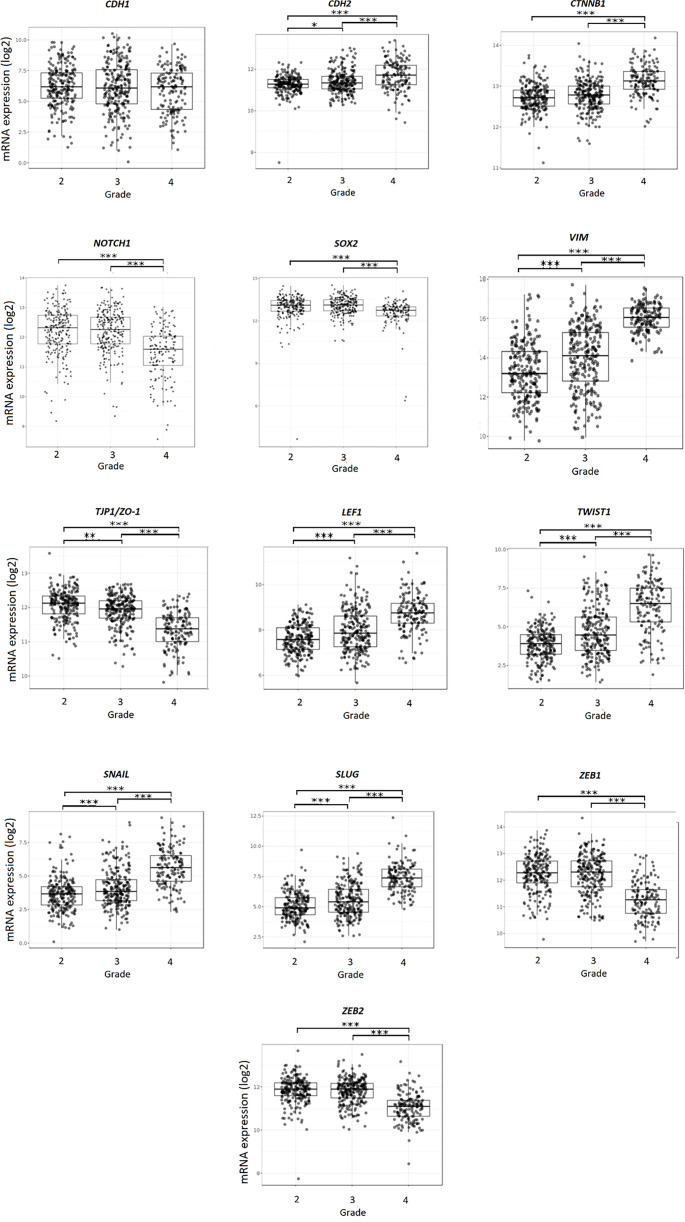
**Gene expression in glioma obtained from GlioVis, TCGA_GBMLGG dataset.** Glioma grades 4, 3, and 2 were included in the study. Results are presented as mean +/− SD. *P < 0.05, **P < 0.01, ***P < 0.001, ***P < 0.0001 (one-way ANOVA with Tukey’s *post hoc* test). ANOVA: Analysis of variance.

In the next part, we analyzed the gene expression *in silico* (results obtained from GlioVis, TCGA_GBMLGG dataset included). From the results, we can observe that *CDH2*, *CTNNB1*, *VIM*, *LEF1*, *TWIST1*, *SNAI1,* and *SNAI2* are overexpressed in GBM (grade 4) vs gliomas grade 3 and 2. *NOTCH1*, *SOX2*, *TJP1/ZO1*, *ZEB1,* and *ZEB2* have higher expression in lower-grade glioma vs GBM (Figure 3).

### Gene expression in GBM

**Figure 4.**
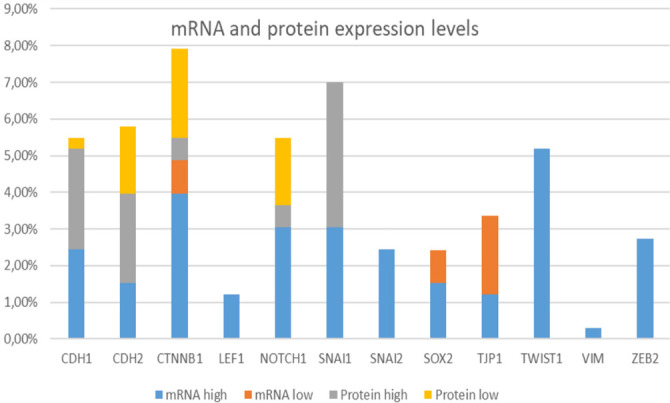
**mRNA and protein expression levels.** Samples from the Glioblastoma Multiforme study (TCGA, Firehose Legacy) were obtained by next-generation sequencing from RNASeq V2 RSEM and downloaded from cBioPortal. TCGA: The Cancer Genome Atlas.

mRNA expression analysis on samples from The *GBM Multiforme* study (TCGA, Firehose Legacy) was obtained by next-generation sequencing from RNASeq V2 RSEM, downloaded from cBioPortal and shown in Figure 4.

A higher level of mRNA expression was noted for the majority of queried genes: *TWIST1, CTNNB1, SNAI1, NOTCH1, ZEB2, SNAI2, CDH1, CDH2*, and *LEF1,* while gene *TJP1/ZO-1* showed a reduced level of mRNA transcript. For *SOX2*, a similar number of samples had elevated mRNA levels as well as decreased. Figure 4 also shows high/low protein expression for several genes.

We also investigated post-transcriptional events by analyzing methylation patterns. We inspected cBioPortal and found that mRNA expression levels were associated with levels of methylation for genes *CDH1* (Spearman: −0.27; *P* ═ 0.0209), *NOTCH1* (Spearman: −0.22; *P* ═ 0.0619), *TJP1/ZO-1* (Spearman: −0.27; *P* ═ 0.0174), *SNAI1* (Pearson: −0.30; *P* ═ 0.0174), *SNAI2* (Pearson: 0.28; *P* ═ 0.0251), and *VIM* (Spearman: 0.25; *P* ═ 0.0496).

### Differential analysis stratified according to *IDH1* status

**Figure 5.**
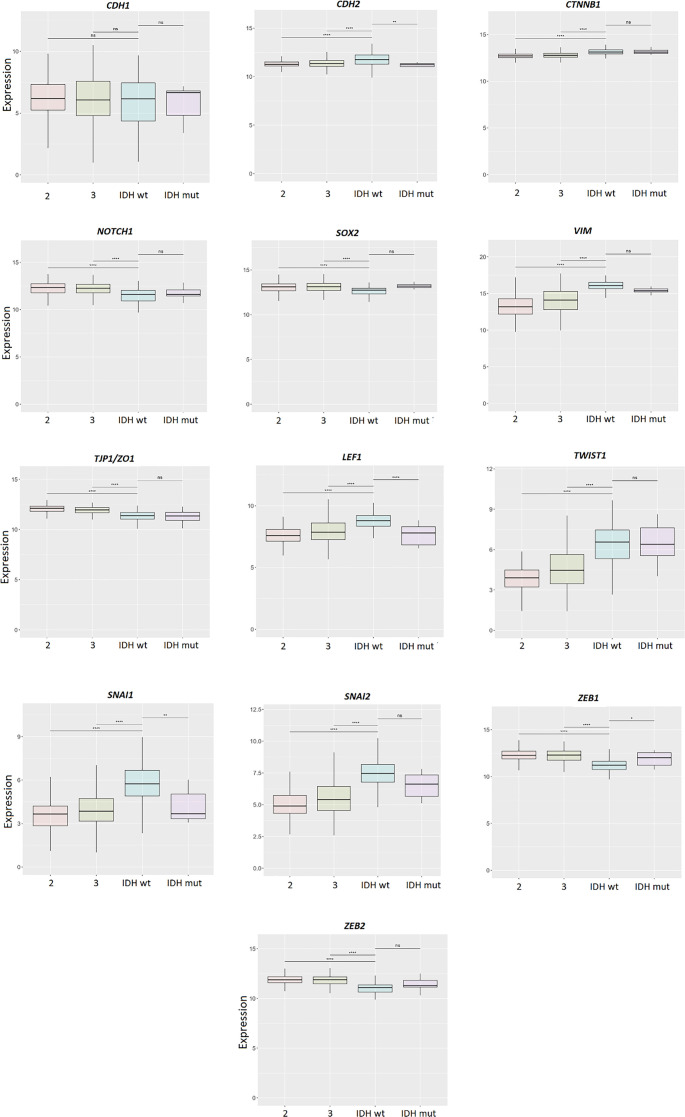
**Differential expression stratified according to *IDH1* status.** Results are presented as mean +/− SD. *P < 0.05, **P < 0.01, ***P < 0.001, ***P < 0.0001 (one-way ANOVA with Tukey’s *post hoc* test). 2, 3, IDH1wt (4), IDH1 mut (4) represent glioma grades. ANOVA: Analysis of variance.

The results of differential expression analysis performed on TCGA_GBMLGG in grade 2, grade 3, and grade 4 (GBM), stratified according to *IDH1* wt and *IDH1* mutations, indicated similar results that were obtained without this stratification (Figure 5). However, when we divided samples for *IDH1* wt/mut, it was demonstrated that genes *CDH2, LEF1, SNAI1,* and *ZEB1* showed significant expression differences between *IDH1* wt and *IDH1* mutant GBM. *CDH2*, *LEF1,* and *SNAI1* had lower expression in *IDH1* mutant samples, while *ZEB1* had significantly higher expression levels in samples harboring *IDH1* mutations (Figure 5).

### GBM subtypes analysis

**Figure 6.**
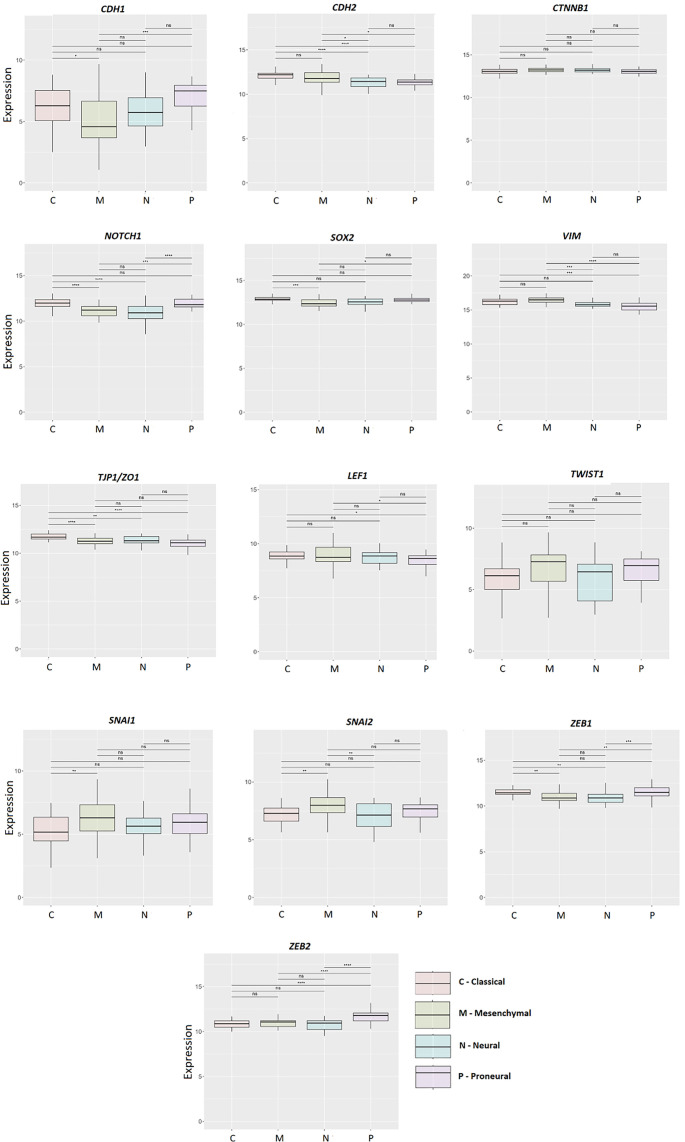
**Differential expression in grade 2, grade 3, and grade 4 (GBM) stratified according to GBM subtypes: Classical, mesenchymal, proneural, and neural.** Results are presented as mean +/− SD. *P < 0.05, **P < 0.01, ***P < 0.001, ***P < 0.0001 (one-way ANOVA with Tukey’s *post hoc* test). ANOVA: Analysis of variance; GBM: Glioblastoma.

The results of differential expression in different GBM subtypes—classical, mesenchymal, proneural, and neural—clearly showed that the *TJP1/ZO-1* gene was associated with the classical subtype, while *ZEB2* was associated with the proneural subtype (Figure 6).

### qRT-PCR validation

**Figure 7.**
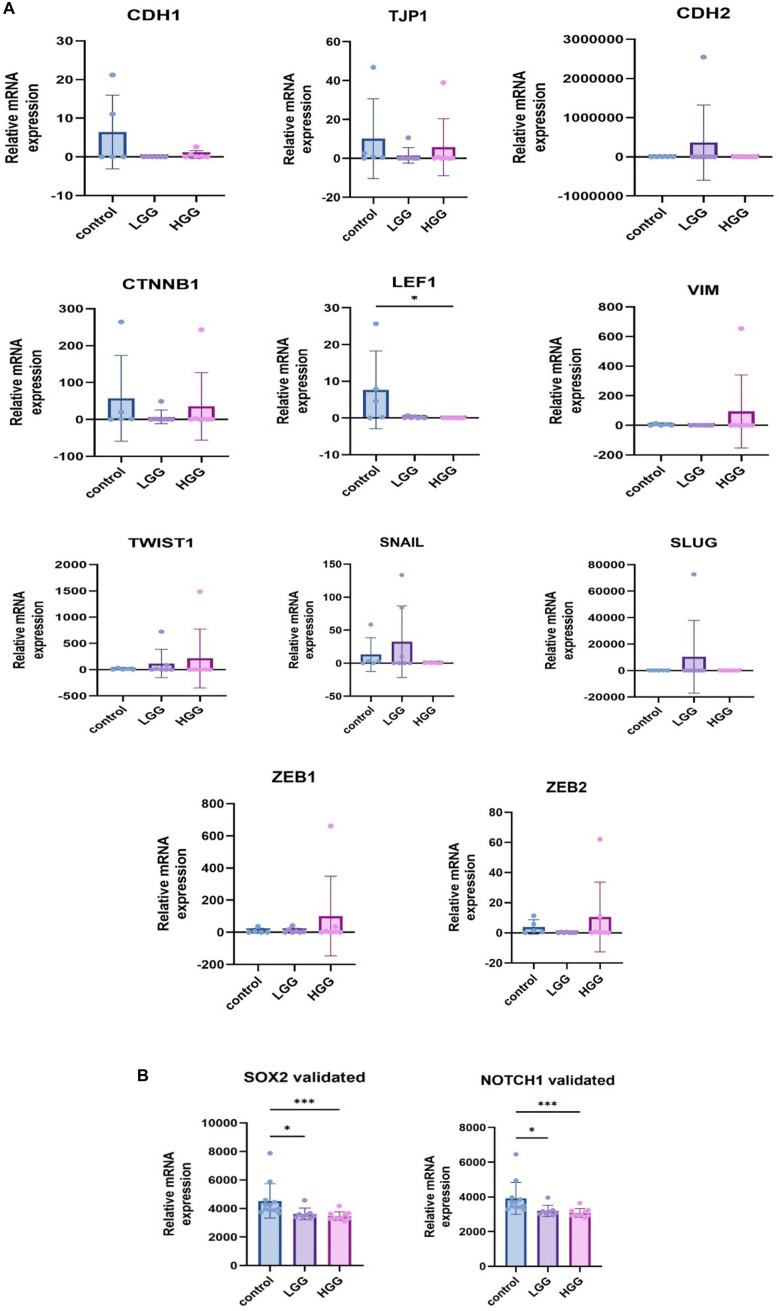
(A) qRT-PCR of analyzed genes; (B) qRT-PCR of *NOTCH1* and *SOX2* genes was validated in additional experiments. Results are presented as mean +/− SD. *P < 0.05, **P < 0.01, ***P < 0.001, ***P < 0.0001. qRT-PCR: Quantitative real-time polymerase chain reaction.

We performed qRT-PCR for all selected genes in HGG, LGG, and normal brain tissues. Except for *LEF1*, *NOTCH1,* and *SOX2*, there was no statistical significance observed in the upregulation or downregulation of the candidate genes. However, the expression levels differed. The levels of *CDH1, LEF1,* and *TJP1/ZO-1,* were lower in both LGG and HGG in comparison to normal controls. *CTNNB1*, *TWIST1, VIM, ZEB1*, and *ZEB2* had higher levels in HGG than LGG, while *SOX2*, *NOTCH1, SNAI1, SNAI2* and *CDH2*, had higher levels in LGG than in HGG (Figure 7A and 7B). When comparing our qRT-PCR results to both databases, we observed that expressions were compatible for the majority of genes (Table 2). *CTNNB1* rose in higher grades, which was compatible with data from GlioVis. Both *NOTCH1* and *SOX2* expression fell in higher grades, which was compatible with GlioVis and cBioPortal. *TJP1/ZO-1* was low in both groups, lower than controls, and this was in accordance with both cBioPortal and GlioVis and was biologically logical. *VIM* and *TWIST1* qRT-PCR levels were concordant with both GlioVis and cBioPortal. qRT-PCR data were partially compatible for *SNAI1, SNAI2*, and *CDH2* in showing higher levels in LGG, but discordant with GlioVis for lower levels in HGG. However, both *ZEB1* and *ZEB2* were rising in higher grades, showing higher levels of expression than controls, which was different from GlioVis. However, *ZEB2* was compatible with GBM high expression reported in cBioPortal. *LEF1* was also not compatible with the databases. qRT-PCR showed *CDH1* levels lower than controls, which is biologically logical. *CDH1* did not show a difference between LGG and HGG, which was compatible with GlioVis (Table S2 shows representative raw Ct values).

**Table 2:** Concordance and discordance of expression data

	***CDH1***	***CDH2***	***TJP1/** **ZO-1***	***CTNNB1***	***LEF1***	***VIM***	***NOTCH1***	***SNAI1***	***SNAI2***	***TWIST1***	***SOX2***	***ZEB1***	***ZEB2***
cBioPortal and GlioVis	dis	con	con	con	con	con	uc	con	con	con	uc	uc	dis
GlioVis and qRT-PCR	con	con/dis	con	con	dis	con	con	con/dis	con/dis	con	con	dis	dis
cBioPortal and qRT-PCR	dis	dis	con	con	dis	con	con	dis	dis	con	con	uc	con

Con: Concordance; dis: Discordance; uc: Unconclusive; LEF1: Lymphoid Enhancer Binding Factor 1; qRT-PCR: Quantitative real-time polymerase chain reaction.

### Survival analyses

**Figure 8.**
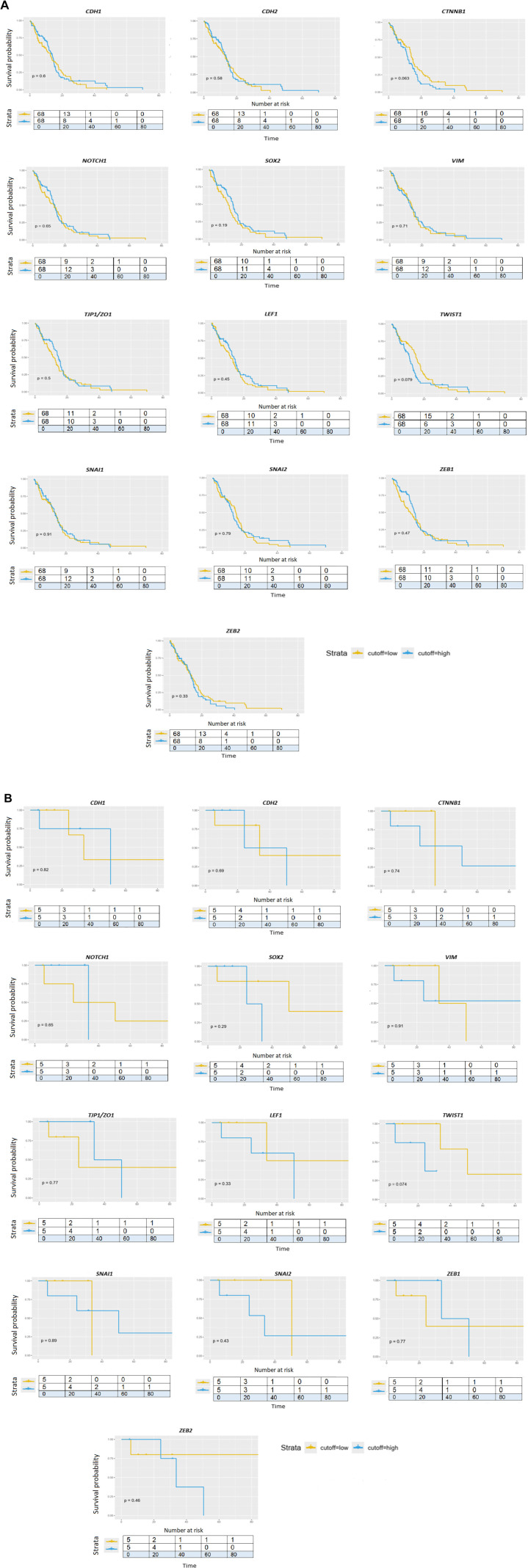
**Age-adjusted survival analysis of GBM patients related to EMT-associated genes.** Kaplan–Meier curves of overall survival (OS) of genes involved in EMT in TCGA_GBMLGG cohort data. (A) GBM *IDH1* WT age-adjusted survival; (B) GBM *IDH1* MUT age-adjusted survival. EMT: Epithelial-to-mesenchymal transition; TCGA: The Cancer Genome Atlas; GBM: Glioblastoma.

In the last part, we aimed to analyze the relationship between gene expression and GBM patient survival. From the results, we can observe that no gene except *TWIST1* is related to survival, while higher expression of *TWIST1* is associated with shorter overall survival (*P* ═ 0.018). Age-adjusted survival analysis showed that in *IDH1* wt GBMs, no gene was associated with worse or better survival with a *P* value lower than 0.05. *TWIST1* had a *P* value of 0.079, while *CTNNB1* had a *P* value of 0.063 (Figure 8A). For GBMs that were *IDH1* mutated, again no gene was associated with worse or better survival; *TWIST1* again reached a value of *P* ═ 0.074. (Figure 8B) (Table 3). The limitation of the survival analyses is that they were not supplemented with outcome modifiers such as treatment variables that may co-vary with gene expression. However, MGMT methylation status was provided in the raw data, and we performed survival analysis of GBM *IDH1* wt adjusted for MGMT methylation status. No gene was associated with worse or better survival.

**Table 3:** Results for survival, GBM subtype, and expression (*in silico* and qRT-PCR) are presented for each gene

**Gene**	**Survival (*IDH1*wt)**	**Survival** **(*IDH1* mut)**	**Subtype**	**Expression in GBM *IDH1* wt compared to Grade 2 and Grade 3 (*in silico*)**	**Expression in GBM *IDH1* wt compared to control (qPCR)**
*CDH1*	-	-	-	-	-
*CDH2*	-	-	-	*↑*	-
*TJP1/ZO-1*	-	-	CLAS	*↓*	-
*CTNNB1*	(+; *P* ═ 0.063)	-	-	*↑*	-
*LEF1*	-	-	-	*↑*	*↓*
*NOTCH1*	-	-	-	*↓*	*↓*
*SNAI1*	-	-	-	*↑*	-
*SNAI2*	-	-	-	*↑*	
*SOX2*	-	-	-	*↓*	*↓*
*TWIST1*	(+; *P* ═ 0.079)	(+; *P* ═ 0.074)	-	*↑*	-
*VIM*	-	-	-	*↑*	-
*ZEB1*	-	-	-	*↓*	-
*ZEB2*	-	-	PN	*↓*	-

CLAS: Classical subtype; PN: Proneural subtype; LEF1: Lymphoid Enhancer Binding Factor 1; qRT-PCR: Quantitative real-time polymerase chain reaction; GBM: Glioblastoma.

### Protein network analysis

**Figure 9.**
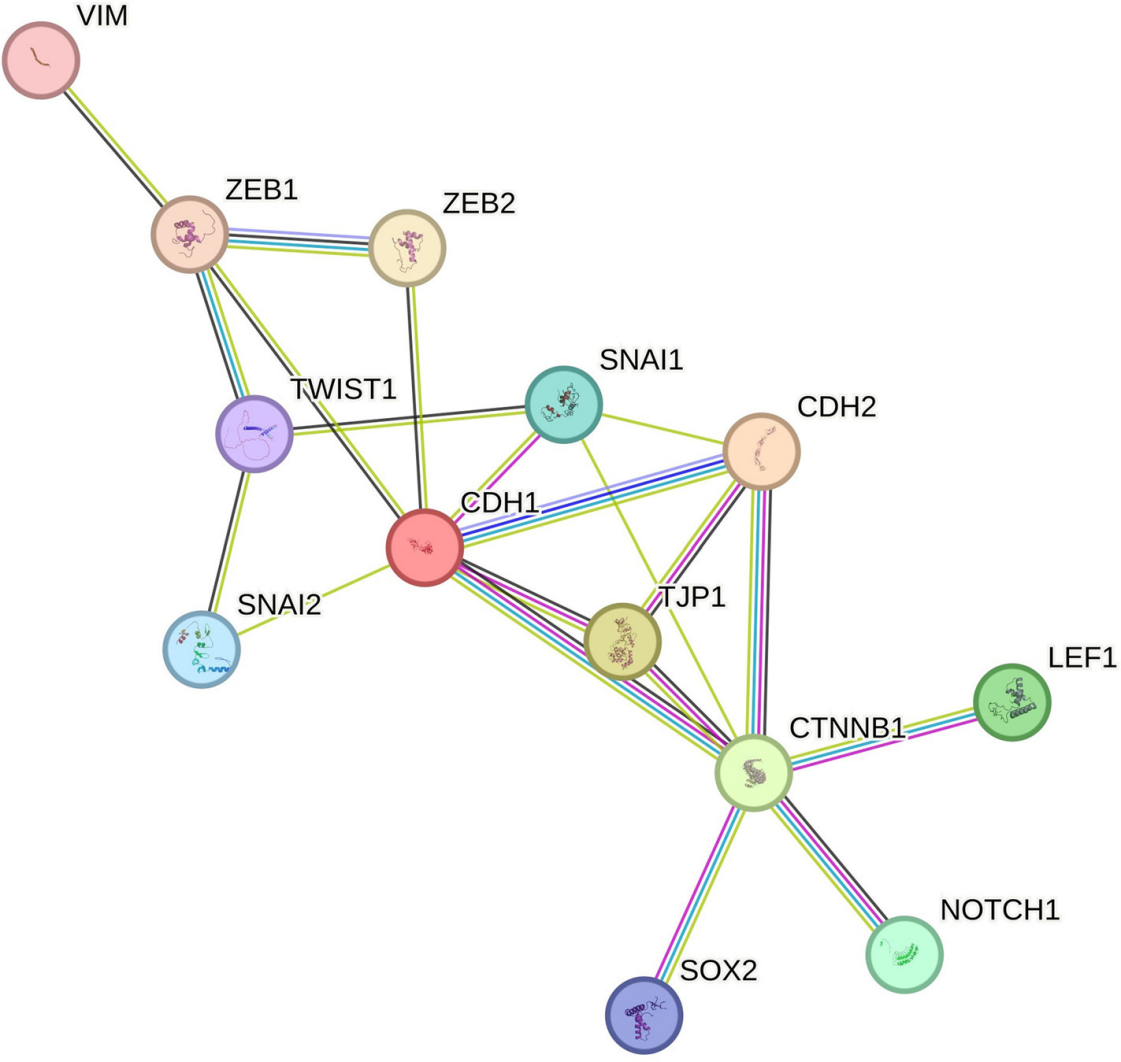
**Protein network of EMT-related genes (STRING tool).** EMT: Epithelial-to-mesenchymal transition.

A network of NOTCH1 and SOX2 was constructed using the STRING tool, showing confidence in the connection. No other interactors were included in the network. The highest confidence (0.9) was applied. From Figure 9, it is obvious that there is a strong interconnection between different EMT genes.

### Enriched pathways

**Figure S1.**
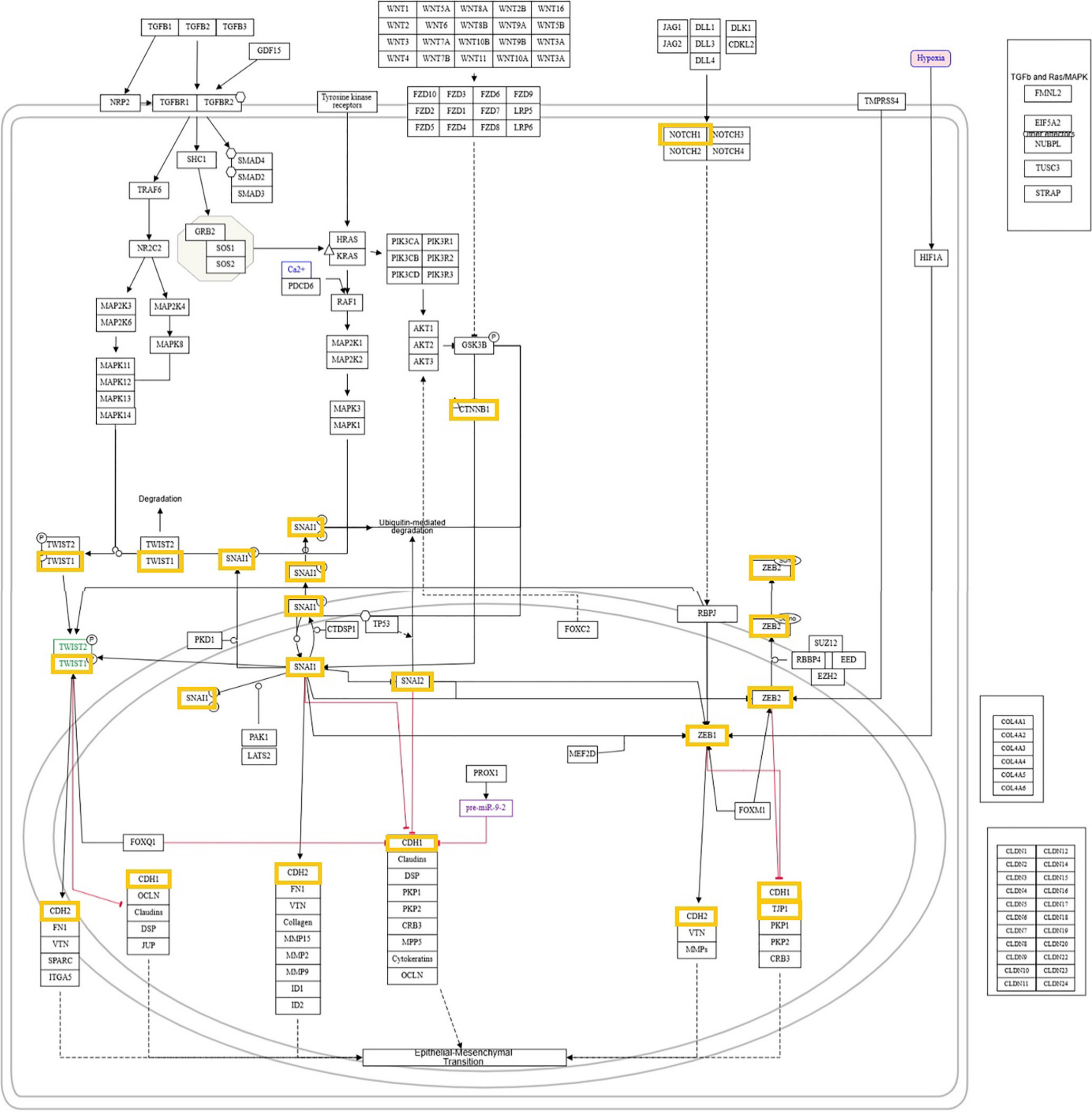
**Pathway enrichment.** Pathway WP4239 (Epithelial-to-mesenchymal transition in colorectal cancer) is presented.

Enrichment analysis indicated pathways where the selected EMT genes are interconnected. The pathways include: WP4239 (EMT in colorectal cancer), WP5097 (CCL18 signaling that led to EMT or migration and invasion), and WP5469 (Hallmark of cancer: metastasis and EMT) (Figure S1).

## Discussion

Transcriptional program switching between epithelial and mesenchymal phenotypes is induced by multiple factors and signaling pathways [32, 33]. A present investigation of genes involved in EMT showed that the frequency and type of their changes were different across human glioma types. The investigation *in silico* included 3143 patients with glial tumors of various malignancy grades from cBioPortal, a public database for interactive exploration of multidimensional datasets in cancer genomics. cBioPortal provides high-quality access to molecular profiles and clinical parameters collected from large-scale cancer genomics projects and experimental studies. This database enables large-scale data processing, statistical analysis, and a graphical overview of changes observed in human tumors from the gene to the protein level.

The first among the queried genes was the primary marker of the epithelial phenotype, E-cadherin, encoded by the *CDH1* gene. This transmembrane glycoprotein is localized in adherens junctions [34]. Reduced expression of E-cadherin is considered one of the main molecular events responsible for EMT [35]. In tumors, it has been assigned the role of a tumor suppressor, whose loss is particularly involved in the mechanisms of invasiveness [36, 37]. Schwechheimer et al. [38] reported a lack of E-cadherin expression in astrocytomas, GBMs, and oligodendrogliomas, which is in accordance with reports on the frequent promoter hypermethylation of this gene. The results of our study showed frequent mutations of *CDH1* associated with higher malignancy grades, out of which seven were characterized as oncogenic, suggesting an increase in invasive potential in more malignant tumors. Another investigated marker of the epithelial phenotype was *TJP1/ZO-1* (TJP1, *Zonula occludens-1*, ZO-1), also known as tight junction protein-1. It encodes a 220 kDa cell membrane protein that acts as a tight junction adapter between the membrane and the actin cytoskeleton [39]. Mutations were also the most common change for TJP1/ZO-1. Higher grades also harbored more mutations compared to lower ones. The expression levels of CDH1 were slightly lower in higher grades, while the *TJP1/ZO-1* transcript was significantly lower in higher grades according to GlioVis. qRT-PCR showed that *CDH1* and *TJP1/ZO-1* levels were lower than controls, indicating a reduced epithelial phenotype in higher-grade gliomas. One of the most important markers of the mesenchymal phenotype is N-cadherin, encoded by the *CDH2* gene [9]. The protein plays a major role in the formation of nervous tissues, but in tumors, N-cadherin enhances the ability of cells to migrate and invade surrounding tissues [40, 41]. We have shown that the *CDH2* gene was most often mutated in anaplastic astrocytoma (grade 3, 4.08%), while amplifications were present in a smaller percentage of diffuse gliomas and GBMs in which deep deletions were also recorded. Chen et al. [42] showed that N-cadherin may serve as a prognostic indicator for overall survival in patients with glioma. By studying TCGA, Chinese Glioma Genome Atlas (CGGA), and Rembrandt databases, *CDH2* expression was identified as significantly higher in grade 4 than in grades 2 (*P* < 0.001) or 3 (*P* < 0.001) [43]. Our investigation showed that mRNA expression levels were significantly different between grades 2 and 4, 3 and 4, and 2 and 3, according to GlioVis, TCGA_GBMLGG. The rise in expression was significantly associated with higher grades, which corroborates the above-mentioned studies. New research shows that there is an abundant expression of the precursor of N-cadherin-proN-cadherin in the cell membrane of most examined gliomas [43].

*NOTCH1* and *SOX2* were genes mostly affected by changes in both LGG and HGG. It has been recognized that both *SOX2* and *NOTCH1* are molecules essential for invasiveness and metastasis. *NOTCH1* is one of the four genes encoding a member of the NOTCH family of signaling receptors [44, 45]. *NOTCH1* is upregulated in malignant tumors, has a central function in progression, and has been shown to promote EMT through Notch ligands named Jagged [46–48]. A highly active Notch signal is observed in glioma stem cells (GSCs) [49, 50]. Furthermore, the low overall survival has been attributed to *NOTCH1* overexpression. Although studies have shown that *NOTCH1* helps to induce EMT in both healthy and neoplastic cells, its role as a marker of the mesenchymal phenotype is still controversial, especially in gliomas, where *NOTCH1* has not yet been elucidated regarding its role in EMT.

The transcription factor SOX2 (*SRY-Box Transcription Factor 2*) is associated with the late stages of EMT. Its intronless gene encodes a member of the high-mobility group box (HMG-box, SOX) family of transcription factors associated with sex-determining region-Y (SRY) [51, 52]. The *SOX2* gene product is required for the maintenance of stem cells in the CNS. Guetta-Terrier et al. [53] showed that *SOX2* was upregulated to reduce the methylation level of the *NOTCH1* promoter and enhance its expression in GSCs [33]. The expression levels of *NOTCH1* in GBM were positively correlated with *SOX2* and *VIM* [33] in their study.

The present investigation indicates that the prevalent type of changes for *NOTCH1* were mutations, while amplifications and deep deletions were rare. There were 193 mutations, of which 72 were characterized as oncogenic drivers. *SOX2,* on the other hand, was predominantly amplified. Survival analyses showed no correlation between *NOTCH1* and *SOX2* expression and the survival of GBM patients. However, the data from GlioVis, TCGA_GBMLGG showed that there was a significant difference in mRNA expression levels for both *NOTCH1* and *SOX2*, where grade 4 had significantly lower expression levels compared to grades 2 and 3. Experimental validation with qRT-PCR showed that both *NOTCH1* and *SOX2* expression levels were falling in higher grades, which confirmed *in silico* results from GlioVis and cBioPortal. A significant decrease in the expression of *NOTCH1* and *SOX2* between control tissue and LGG and HGG was established.

In their work, Song et al. [54] showed that CDH1/β-catenin and Notch-1/Akt signaling pathways are targeted in glioma. Several components of the Notch pathway, including *NOTCH1*, are highly expressed at the invasive edges of tumors, and the same can be said for the EMT marker vimentin. Notch also regulates the transcription of ZEB, Snail, and Slug, which repress E-cadherin and induce vimentin expression. Here, we showed that the *NOTCH1* gene was highly mutated in both LGG and HGG gliomas, which may indicate that such alterations happen early and are constant throughout the stages of glioma progression.

Defective activation of the Wnt signaling pathway has been detected in various cancers, including glioma [55], and nuclear accumulation of β-catenin is positively correlated with metastasis and recurrence, resulting in poor clinical outcomes [56] characterizing β-catenin as a marker of the mesenchymal phenotype. The protein is part of the complex that makes up adherens junctions, where it anchors the actin cytoskeleton [57]. In addition, β-catenin is also the main signaling molecule of the Wnt pathway. Prior studies report higher β-catenin and C-myc activity in relapsed glioma than in the primary tumor [58]. Our results on the *CTNNB1* gene (β-catenin) showed that mutations were specifically frequent in anaplastic gliomas. Additionally, the total mutational burden was higher in HGG. When querying its mRNA expression levels, they were significantly higher in grade 4 tumors compared to grades 2 and 3, which indicates that its excessive expression has oncogenic properties. qRT-PCR showed that *CTNNB1* rose in higher grades, which was compatible with data from GlioVis. β-catenin’s partner in the transcription regulation of the Wnt signaling is LEF1. This transcription factor contains a high-mobility group (HMG) DNA-binding domain and is generally excessively expressed in malignant tumors. LEF1 promotes mesenchymal cell properties in EMT [59, 60] and was significantly associated with the overall survival of glioma patients. Reports indicate that reduced expression of LEF1 inhibited cell migration, invasion, and EMT in GBM cells [61]. In our study, the changes in the *LEF1* gene were confined to diffuse glioma and GBM. Its mRNA expression levels were significantly higher in grades 3 and 4 compared to grade 2, which was not supported by our qRT-PCR results.

A type-3 intermediate filament protein, vimentin, encoded by *VIM,* is another well-known mesenchymal marker responsible for cytoskeletal interactions. It functions as an organizer of several other key proteins involved in cell attachment, migration, and signaling [62]. Glioma types sustained different changes,—in GBM, the gene was mutated, and in diffuse gliomas, it was predominantly amplified. Higher levels of mRNA expression were noted, and significant differences in the expression levels were recorded between grades 2 and 3, 2 and 4, and 3 and 4, where higher mRNA levels were associated with higher grades according to GlioVis, which was corroborated by qRT-PCR. Generally, the activation of EMT-TFs leads to the decreased expression of epithelial markers and increased expression of mesenchymal markers. They all bind to the E-box, the *cis*-regulatory element of the *CDH1* gene, and thus act as repressors of E-cadherin expression [63, 64]. Our study showed that genes for transcription factors from the protein families SNAIL, ZEB, and TWIST were mostly altered by mutations and amplifications in HGG. It is known that the transcriptional repressor TWIST1, through binding to E-cadherin’s promoter or by inducing SNAI1, promotes chromosomal instability, angiogenesis, invasion, metastasis, and resistance to chemotherapy [65, 66]. SNAI1 (SNAIL) and SNAI2 (SLUG) are zinc-finger transcription factors that maintain a mesenchymal and undifferentiated phenotype by controlling invasive characteristics [57, 59, 67, 68]. ZEB1 and ZEB2 are two closely related EMT transcriptional regulators of the Zinc Finger E-box Binding Homeobox family. The role of both ZEBs is to promote EMT, tumor progression, and metastasis through E-cadherin downregulation. It has also been documented that their overexpression has been found in several cancers and that they are responsible for therapy resistance [69–71]. It is known that ZEB1/2 are highly regulated in the early stage of hybrid EMT and that their high level is maintained in mesenchymal cell populations [69]. *ZEB2* has previously been confirmed to be associated with the malignant phenotype of glioma [72], and the expression level of *ZEB1* was significantly increased in glioma tissues compared to normal brain tissues, being positively correlated with WHO glioma classification [73]. In this investigation, we have shown that *ZEB1* and *ZEB2* mutations were more common in HGGs and that qRT-PCR results showed higher transcript levels in HGG compared to LGG and normal brain. However, GlioVis reported significant downregulation of both transcripts in grade 4 tumors compared to grade 3, which was contrary to our qRT-PCR results. Furthermore, we have shown that *TWIST1* was altered predominantly by amplifications in GBM and diffuse glioma. Additionally, its transcript was significantly higher in higher glioma grades, which was confirmed by qRT-PCR validation. *TWIST1* was related to shorter overall survival (*P* ═ 0.018), and for age-adjusted survival in *IDH1* wt and mutant GBMs, *TWIST1* was again indicated as almost significant (*P* ═ 0.079).

It was recently reported that the transcriptional repressors of the snail family, *SNAI1* and *SNAI2,* play a role in the acquisition and increase of invasiveness in malignant gliomas [67, 68]. It has been shown that *SNAI1* induces EMT through the expression of EMT markers. *SNAI2* expression was increased in GBMs compared to healthy brain tissue [74, 75]. *SNAI1* and *SNAI2* transcript levels rose in higher grades according to GlioVis. *SNAI1* protein levels were also high in GBM according to cBioPortal. qRT-PCR data were partially compatible with GlioVis for *SNAI1* and *SNAI2,* for showing higher levels in LGG when compared to controls, but discordant for lower levels in HGG.

Potential explanations for the evidenced discrepancies could be attributed to post-transcriptional regulation, tumor heterogeneity, or technical limitations in sample processing. We also investigated post-transcriptional regulation by analyzing methylation patterns to provide additional insights into the underlying disease mechanisms and patterns. mRNA expression levels were associated with levels of methylation for genes *CDH1, NOTCH1, TJP1/ZO-1, SNAI1, SNAI2*, and *VIM*.

Differential expression analysis stratified by *IDH1* mutations showed that *IDH1* mutant samples had significantly lower *CDH2, LEF1,* and *SNAI1* expression, while *ZEB1* was significantly higher. Gene expression in different GBM subtypes showed that the *TJP1/ZO-1* gene was associated with the classical subtype, while *ZEB2* was associated with the proneural one.

Our study showed that all genes representative of EMT are mutated or altered in certain ways in gliomas and that some show a marked involvement and are associated with a higher grade of glioma. Statistical analysis showed differences when looking at the overall changes in the pathohistological glioma types. Thus, astrocytomas harbored more changes in selected genes, i.e., all genes were affected. Furthermore, the accumulation of changes from diffuse gliomas to GBM is visible in all examined genes, where changes in the *NOTCH1* and *SOX2* genes were most pronounced. Our findings could be connected to the concept of hybrid EMT, a state in which tumor cells have both mesenchymal and epithelial features, making them especially flexible in adapting to the new tumor microenvironment [4, 5, 8]. Observed gene expression patterns (e.g., co-occurrence of epithelial and mesenchymal markers) support partial EMT in gliomas. Several recent studies provide evidence of EMT plasticity in glioma cells. A paper reports on cancer stemness-associated genes in gliomas determined by their relative mRNA expression [76].

We have to mention another possible mechanism contributing to EMT plasticity, for instance, RNA interference [77]. A study by Qu et al. [78] indicated that Hsa-miR-196a-5p overexpression was associated with the clinical malignant biological behavior of glioma.

Immune cell infiltration is also vital for the glioma microenvironment [79, 80]. Important research was conducted on proinflammatory-related molecules as promising immune biomarkers significantly associated with clinical indicators of malignant progression in glioma patients. Tumor-infiltrating immune cells are strongly associated with tumorigenesis and progression; for instance, the transcription factor CASZ1 [81, 82] was significantly upregulated in gliomas and was related to EMT signaling. At present, there are many studies on the latest prognostic biomarkers and targets of glioma [76–83]. The plasticity of hybrid EMT allows cancer cells to adapt to environmental stress during malignant progression. Our validation by qRT-PCR demonstrated that the levels of *CDH1, LEF1,* and *TJP1/ZO-1,* were lower in both LGG and HGG in comparison to normal controls. *CTNNB1*, *TWIST1, VIM, ZEB1*, and *ZEB2* had higher levels in HGG than LGG, while *SOX2*, *NOTCH1, SNAI1, SNAI2,* and *CDH2* had higher levels in LGG than in HGG.

It is also important to highlight that the enrichment analysis indicated pathways where the genes we investigated are interconnected. The pathways that emerged as significantly enriched were two epithelial to mesenchymal transition pathways and CCL18 signaling that led to several EMT pathways or migration and invasiveness.

Although our current study is primarily bioinformatics-based, experimental validation would certainly advance the causality of this research direction. In order to functionally validate key drivers, *NOTCH1* and *SOX2*, prospective future studies based on strategies of knockdown experiments, invasion, and migration assays need to be additionally conducted to confirm the causal role of these genes in EMT phenotypes. For that purpose, GBM cell lines (e.g., U87 and LN229) should be used for knockdown experiments targeting *NOTCH1* and *SOX2* via siRNA transfection. Cells will be transfected with siNOTCH1 and siSOX2, alongside a non-targeting siRNA control, using Lipofectamine or equivalent transfection reagents following the manufacturer’s protocol. Following knockdown, EMT-associated phenotypes need to be assessed, including proliferation, migration, and invasion. Migration and invasion will be evaluated using Transwell chamber assays, while proliferation will be assessed using MTT or BrdU incorporation assays. Additional research involving functional experiments is needed to establish causality between gene alterations and EMT phenotypes and confirm the knowledge that this work has brought.

## Conclusion

This comprehensive study shows that genes associated with mesenchymal transition, *CDH1*, *CTNNB1*, *TJP1*, *TWIST1*, *SOX2*, *ZEB1,* and *ZEB2,* have higher frequencies of alterations in HGG vs LGG, indicating a shift toward a more invasive phenotype. Of those genes, *CTNNB1*, *TWIST1, VIM, ZEB1*, and *ZEB2* had higher expression levels in HGG than in LGG, while *SOX2*, *NOTCH1, SNAI1, SNAI2,* and *CDH2* had higher levels in LGG. In comparison to controls, low levels of transcripts of markers of epithelial phenotype, *CDH1* and *TJP1* were recorded. Overall, *NOTCH1* and *SOX2*, key regulators of EMT, are most frequently altered in both HGG and LGG, indicating their universal role across different glioma tumors. These results provide valuable insights into the molecular differences between LGG and HGG, emphasizing the potential relevance of EMT-related genes in glioma biology and patient prognosis.

## Supplemental data

**Table S1:** Primer sequences used for qRT-PCR

**Gene**	**Forward primer**	**Reverse primer**
*ACTB*	GAAGAGCTACGAGCTGCCTGA	CCACGTCACACTTCATGATGG
*CDH1*	GCCTCCTGAAAAGAGAGTGGAAG	TGGCAGTGTCTCTCCAAATCCG
*CDH2*	CCTCCAGAGTTTACTGCCATGAC	GTAGGATCTCCGCCACTGATTC
*CTNNB1*	CACAAGCAGAGTGCTGAAGGTG	GATTCCTGAGAGTCCAAAGACAG
*LEF1*	CTACCCATCCTCACTGTCAGTC	GGATGTTCCTGTTTGACCTGAGG
*NOTCH1*	GGTGAACTGCTCTGAGGAGATC	GGATTGCAGTCGTCCACGTTGA
*SNAIL*	TGCCCTCAAGATGCACATCCGA	GGGACAGGAGAAGGGCTTCTC
*SLUG*	ATCTGCGGCAAGGCGTTTTCCA	GAGCCCTCAGATTTGACCTGTC
*SOX2*	GCTACAGCATGATGCAGGACCA	TCTGCGAGCTGGTCATGGAGTT
*TJP1/ZO-1*	GTCCAGAATCTCGGAAAAGTGCC	CTTTCAGCGCACCATACCAACC
*TWIST1*	GCCAGGTACATCGACTTCCTCT	TCCATCCTCCAGACCGAGAAGG
*VIM*	AGGCAAAGCAGGAGTCCACTGA	ATCTGGCGTTCCAGGGACTCAT
*ZEB1*	GGCATACACCTACTCAACTACGG	TGGGCGGTGTAGAATCAGAGTC
*ZEB2*	AATGCACAGAGTGTGGCAAGGC	CTGCTGATGTGCGAACTGTAGG

LEF1: Lymphoid Enhancer Binding Factor 1; qRT-PCR: Quantitative real-time polymerase chain reaction.

**Table S2.** Crude qRT-PCR data. qRT-PCR: Quantitative real-time polymerase chain reaction.

Supplementary data are available at the following link: https://www.bjbms.org/ojs/index.php/bjbms/article/view/12598/3958.

## Data Availability

All data generated or analyzed during this study are included in this published article.

## References

[1] Chen T, You Y, Jiang H, Wang ZZ (2017). Epithelial–mesenchymal transition (EMT): a biological process in the development, stem cell differentiation, and tumorigenesis. J Cell Physiol.

[2] Brabletz S, Schuhwerk H, Brabletz T, Stemmler MP (2021). Dynamic EMT: a multi-tool for tumor progression. EMBO J.

[3] Hanahan D, Weinberg RA (2011). Hallmarks of cancer: the next generation. Cell.

[4] Saitoh M (2018). Involvement of partial EMT in cancer progression. J Biochem.

[5] Pastushenko I, Blanpain C (2019). EMT transition states during tumor progression and metastasis. Trends Cell Biol.

[6] Nieto MA (2011). The ins and outs of the epithelial to mesenchymal transition in health and disease. Annu Rev Cell Dev Biol.

[7] Loh CY, Chai JY, Tang TF, Wong WF, Sethi G, Shanmugam MK (2019). The E-cadherin and N-cadherin switch in epithelial-to-mesenchymal transition: signaling, therapeutic implications, and challenges. Cells.

[8] Aiello NM, Kang Y (2019). Context-dependent EMT programs in cancer metastasis. J Exp Med.

[9] Mrozik KM, Blaschuk OW, Cheong CM, Zannettino ACW, Vandyke K (2018). N-cadherin in cancer metastasis, its emerging role in haematological malignancies and potential as a therapeutic target in cancer. BMC Cancer.

[10] Xing Y, He M, Su Z, Yasinjan F, Liu J, Wang H (2022). Emerging trends and research foci of epithelial–mesenchymal transition in gliomas: a scientometric analysis and review. Front Oncol.

[11] Chen R, Smith-Cohn ML, Cohen A, Colman H (2017). Glioma subclassifications and their clinical significance. Neurotherapeutics.

[12] Louis DN, Perry A, Wesseling P, Brat DJ, Cree IA, Figarella-Branger D (2021). The 2021 WHO classification of tumors of the central nervous system: a summary. Neuro Oncol.

[13] Park YW, Vollmuth P, Foltyn-Dumitru M, Sahm F, Ahn SS, Chang JH (2023). The 2021 WHO classification for gliomas and implications on imaging diagnosis: part 1-key points of the fifth edition and summary of imaging findings on adult-type diffuse gliomas. J Magn Reson Imaging.

[14] Cerami E, Gao J, Dogrusoz U, Gross BE, Sumer SO, Aksoy BA (2012). The cBio cancer genomics portal: an open platform for exploring multidimensional cancer genomics data. Cancer Discov.

[15] Gao J, Aksoy BA, Dogrusoz U, Dresdner G, Gross B, Sumer SO (2013). Integrative analysis of complex cancer genomics and clinical profiles using the cBioPortal. Sci Signal.

[16] Barthel FP, Johnson KC, Varn FS, Moskalik AD, Tanner G, Kocakavuk E (2019 Dec). Longitudinal molecular trajectories of diffuse glioma in adults. Nature.

[17] Jonsson P, Lin AL, Young RJ, DiStefano NM, Hyman DM, Li BT (2019 Sep 15). Genomic correlates of disease progression and treatment response in prospectively characterized gliomas. Clin Cancer Res.

[18] Johnson BE, Mazor T, Hong C, Barnes M, Aihara K, McLean CY (2014 Jan 10). Mutational analysis reveals the origin and therapy-driven evolution of recurrent glioma. Science.

[19] Ceccarelli M, Barthel FP, Malta TM, Sabedot TS, Salama SR, Murray BA (2016 Jan 28). Molecular profiling reveals biologically discrete subsets and pathways of progression in diffuse glioma. Cell.

[20] Vaubel RA, Tian S, Remonde D, Schroeder MA, Mladek AC, Kitange GJ (2020 Mar 1). Genomic and phenotypic characterization of a broad panel of patient-derived xenografts reflects the diversity of glioblastoma. Clin Cancer Res.

[21] Wang LB, Karpova A, Gritsenko MA, Kyle JE, Cao S, Li Y (2021 Apr 12). Clinical proteomic tumor analysis consortium. proteogenomic and metabolomic characterization of human glioblastoma. Cancer Cell.

[22] Zhao J, Chen AX, Gartrell RD, Silverman AM, Aparicio L, Chu T (2019 Mar). Immune and genomic correlates of response to anti-PD-1 immunotherapy in glioblastoma. Nat Med.

[23] The cBioPortal for cancer genomics [Internet]. https://www.cbioportal.org/results/comparison?cancer_study_list=difg_glass_2019%2Cglioma_mskcc_2019%2Clgggbm_tcga_pub%2Cgbm_mayo_pdx_sarkaria_2019%2Cgbm_cptac_2021%2Cgbm_columbia_2019%2Cgbm_tcga%2Clgg_ucsf_2014&Z_SCORE_THRESHOLD=2.0&RPPA_SCORE_THRESHOLD=2.0&profileFilter=mutations%2Cstructural_variants%2Cgistic%2Ccna&case_set_id=all&gene_list=CDH1%250ACDH2%250ACTNNB1%250ALEF1%250ANOTCH1%250ASNAI1%250ASNAI2%250ASOX2%250ATJP1%250ATWIST1%250AVIM%250AZEB1%250AZEB2&geneset_list=%20&tab_index=tab_visualize&Action=Submit&comparison_subtab=survival&comparison_selectedGroups=%5B%22CDH1%22%2C%22CDH2%22%2C%22CTNNB1%22%2C%22LEF1%22%2C%22NOTCH1%22%2C%22SNAI1%22%5D.

[24] Bowman RL, Wang Q, Carro A, Verhaak RG, Squatrito M (2017). GlioVis data portal for visualization and analysis of brain tumor expression datasets. Neuro Oncol.

[25] Li B, Dewey CN (2011). RSEM: accurate transcript quantification from RNA-Seq data with or without a reference genome. BMC Bioinform.

[26] Hoadley KA, Yau C, Hinoue T, Wolf DM, Lazar A, Drill E (2018). Cell-of-origin patterns dominate the molecular classification of 10,000 tumors from 33 types of cancer. Cell.

[27] Jovčevska I, Zupanec N, Urlep Ž, Vranič A, Matos B, Stokin CL (2017). Differentially expressed proteins in glioblastoma multiforme identified with a nanobody-based anti-proteome approach and confirmed by OncoFinder as possible tumor-class predictive biomarker candidates. Oncotarget.

[28] Bustin SA, Benes V, Garson JA, Hellemans J, Huggett J, Kubista M (2009 Apr). The MIQE guidelines: minimum information for publication of quantitative real-time PCR experiments. Clin Chem.

[29] Livak KJ, Schmittgen TD (2001). Analysis of relative gene expression data using real-time quantitative PCR and the 2(-Delta Delta C(T)) method. Methods (San Diego, Calif.).

[30] Szklarczyk D, Nastou K, Koutrouli M, Kirsch R, Mehryary F, Hachilif R (2025 Jan 6). The STRING database in 2025: protein networks with directionality of regulation. Nucl Acids Res.

[31] Szklarczyk D, Kirsch R, Koutrouli M, Nastou K, Mehryary F, Hachilif R (2023). The STRING database in 2023:protein–protein association networks and functional enrichment analyses for any sequenced genome of interest. Nucl Acids Res.

[32] Sinha D, Saha P, Samanta A, Bishayee A (2020). Emerging concepts of hybrid epithelial-to-mesenchymal transition in cancer progression. Biomolecules.

[33] Wu H, Wei M, Li Y, Ma Q, Zhang H (2022). Research progress on the regulation mechanism of key signal pathways affecting the prognosis of glioma. Front Mol Neurosci.

[34] Haerinck J, Berx G (2021). Partial EMT takes the lead in cancer metastasis. Dev Cell.

[35] Uhlmann K, Rohde K, Zeller C, Szymas J, Vogel S, Marczinek K (2003). Distinct methylation profiles of glioma subtypes. Int J Cancer.

[36] Pećina-Šlaus N (2003). Tumor suppressor gene E-cadherin and its role in normal and malignant cells. Cancer Cell Int.

[37] Venhuizen JH, Jacobs FJC, Span PN, Zegers MM (2020). P120 and E-cadherin: double-edged swords in tumor metastasis. Semin Cancer Biol.

[38] Schwechheimer K, Zhou L, Birchmeier WE (1998). Cadherin in human brain tumours: loss of immunoreactivity in malignant meningiomas. Virchows Arch.

[39] Runkle EA, Mu D (2013). Tight junction proteins: from barrier to tumorigenesis. Cancer Lett.

[40] Hazan RB, Qiao R, Keren R, Badano I, Suyama K (2004). Cadherin switch in tumor progression. Ann N Y Acad Sci.

[41] Dongre A, Weinberg RA (2019). New insights into the mechanisms of epithelial–mesenchymal transition and implications for cancer. Nat Rev Mol Cell Biol.

[42] Chen Q, Cai J, Jiang C (2018). CDH2 expression is of prognostic significance in glioma and predicts the efficacy of temozolomide therapy in patients with glioblastoma. Oncol Lett.

[43] Xiong Y, Liu L, Zhu S, Zhang B, Qin Y, Yao R (2017). Precursor N-cadherin mediates glial cell line-derived neurotrophic factor-promoted human malignant glioma. Oncotarget.

[44] Wang Z, Li Y, Banerjee S, Sarkar FH (2009). Emerging role of Notch in stem cells and cancer. Cancer Lett.

[45] Shi Q, Xue C, Zeng Y, Yuan X, Chu Q, Jiang S (2024). Notch signaling pathway in cancer: from mechanistic insights to targeted therapies. Signal Transduct Target Ther.

[46] Timmerman LA, Grego-Bessa J, Raya A, Bertrán E, Pérez-Pomares JM, Díez J (2004). Notch promotes epithelial-mesenchymal transition during cardiac development and oncogenic transformation. Genes Dev.

[47] Zavadil J, Cermak L, Soto-Nieves N, Bottinger EP (2004). Integration of TGF-beta/Smad and Jagged1/Notch signalling in epithelial-to-mesenchymal transition. EMBO J.

[48] Zhang Q, Wang J, Zhang J, Wang Y, Wang Y, Liu F (2024). Cancer-associated fibroblasts-induced remodeling of tumor immune microenvironment via Jagged1 in glioma. Cell Signal.

[49] Yi L, Zhou X, Li T, Liu P, Hai L, Tong L (2019). Notch1 signaling pathway promotes invasion, self-renewal and growth of glioma initiating cells via modulating chemokine system CXCL12/CXCR4. J Exp Clin Cancer Res.

[50] Zhao G, Deng Z, Li X, Wang H, Chen G, Feng M (2023). Targeting EZH2 regulates the biological characteristics of glioma stem cells via the Notch1 pathway. Exp Brain Res.

[51] Xu YR, Yang WX (2017). SOX-mediated molecular crosstalk during the progression of tumorigenesis. Semin Cell Dev Biol.

[52] Kim O, Sergi Z, Yu G, Yamamoto K, Quezado M, Abdullaev Z (2024). A patient-derived cell model for malignant transformation in IDH-mutant glioma. Acta Neuropathol Commun.

[53] Guetta-Terrier C, Karambizi D, Akosman B, Zepecki JP, Chen JS, Kamle S (2023). Chi3l1 is a modulator of glioma stem cell states and a therapeutic target in glioblastoma. Cancer Res.

[54] Song H, Zhang Y, Liu N, Zhao S, Kong Y, Yuan L (2016). miR-92a-3p exerts various effects in glioma and glioma stem-like cells specifically targeting CDH1/β-catenin and Notch-1/Akt signaling pathways. Int J Mol Sci.

[55] Pećina-Šlaus N, Kafka A, Tomas D, Marković L, Okštajner PK, Sukser V (2014). Wnt signaling transcription factors TCF-1 and LEF-1 are upregulated in malignant astrocytic brain tumors. Histol Histopathol.

[56] Du L, Lee J-H, Jiang H, Wang C, Wang S, Zheng Z (2020). β-Catenin induces transcriptional expression of PD-L1 to promote glioblastoma immune evasion. J Exp Med.

[57] Yang J, Antin P, Berx G, Blanpain C, Brabletz T, Bronner M (2022). Guidelines and definitions for research on epithelial–mesenchymal transition. Nat Rev Mol Cell Biol.

[58] Yu X, Xiao F, Wei Y, Miao L, Zhang W, Zhang X (2022). Elevated β-catenin and C-myc promote malignancy, relapse, and indicate poor prognosis in patients with relapsed glioma. J Cancer Res Ther.

[59] Zirkel A, Lederer M, Stohr N, Pazaitis N, Huttelmaier S (2013). IGF2BP1 promotes mesenchymal cell properties and migration of tumor-derived cells by enhancing the expression of LEF1 and SNAI2 (SLUG). Nucl Acids Res.

[60] Zhou X, Li X, Wang R, Hua D, Sun D, Yu L (2022). Recruitment of LEF1 by Pontin chromatin modifier amplifies TGFBR2 transcription and activates TGFβ/SMAD signalling during gliomagenesis. Cell Death Dis.

[61] Min R-Q, Ma Q (2020). MicroRNA-381 inhibits metastasis and epithelial-mesenchymal transition of glioblastoma cells through targeting LEF1. Eur Rev Med Pharmacol Sci.

[62] Zottel A, Novak M, Šamec N, Majc B, Colja S, Katrašnik M (2023). Anti-Vimentin nanobody decreases glioblastoma cell invasion in vitro and in vivo. Cancers (Basel).

[63] Das V, Bhattacharya S, Chikkaputtaiah C, Hazra S, Pal M (2019). The basics of epithelial-mesenchymal transition (EMT): a study from a structure, dynamics, and functional perspective. J Cell Physiol.

[64] Saitoh M (2023). Transcriptional regulation of EMT transcription factors in cancer. Semin Cancer Biol.

[65] Mikheeva SA, Mikheev AM, Petit A, Beyer R, Oxford RG, Khorasani L (2010). TWIST1 promotes invasion through mesenchymal change in human glioblastoma. Mol Cancer.

[66] Mikheev AM, Mikheeva SA, Severs LJ, Funk CC, Huang L, McFaline-Figueroa JL (2018). Targeting TWIST1 through loss of function inhibits tumorigenicity of human glioblastoma. Mol Oncol.

[67] Han S-P, Kim J-H, Han M-E, Sim H-E, Kim K-S, Yoon S (2011). SNAI1 is involved in the proliferation and migration of glioblastoma cells. Cell Mol Neurobiol.

[68] Kaji T, Arito M, Tsutiya A, Sase T, Onodera H, Sato T (2019). Layilin enhances the invasive ability of malignant glioma cells via SNAI1 signaling. Brain Res.

[69] Korpal M, Lee ES, Hu G, Kang Y (2008). The miR-200 family inhibits epithelial-mesenchymal transition and cancer cell migration by direct targeting of E-cadherin transcriptional repressors ZEB1 and ZEB2. J Biol Chem.

[70] Baqai N, Amin R, Fatima T, Ahmed Z, Faiz N (2024). Expression profiling of EMT transcriptional regulators ZEB1 and ZEB2 in different histopathological grades of oral squamous cell carcinoma patients. Curr Genomics.

[71] Sun Y, Guo G, Zhang Y, Chen X, Lu Y, Hong R (2024). IKBKE promotes the ZEB2-mediated EMT process by phosphorylating HMGA1a in glioblastoma. Cell Signal.

[72] Zeng Y, Que T, Lin J, Zhan Z, Xu A, Wu Z (2021 Jan 5). Oncogenic ZEB2/miR-637/HMGA1 signaling axis targeting vimentin promotes the malignant phenotype of glioma. Mol Ther Nucl Acids.

[73] Sun Y, Jiang Y, Wang Y, Yu P, Su X, Song Y (2022). The epithelial-mesenchymal transition of glioma cells promotes tissue factor expression via the miR200a/ZEB1 axis. Brain Res.

[74] Yang HW, Menon LG, Black PM, Carroll RS, Johnson MD (2010). SNAI2/Slug promotes growth and invasion in human gliomas. BMC Cancer.

[75] Kwon R-Y, Han M-E, Kim Y-J, Kim Y-H, Kim J-Y, Liu L (2017). Roles of zinc-fingers and homeoboxes 1 during the proliferation, migration, and invasion of glioblastoma cells. Tumour Biol.

[76] Qu S, Huang J, Liu J, Wang H (2020). Prognostic significance of cancer stemness-associated genes in patients with gliomas. Clin Transl Med.

[77] Hu Z, Rong Y, Li S, Qu S, Huang S (2020). Upregulated histone deacetylase 6 associates with malignant progression of melanoma and predicts the prognosis of patients. Cancer Manag Res.

[78] Qu S, Qiu O, Huang J, Liu J, Wang H (2021). Upregulation of hsa-miR-196a-5p is associated with MIR196A2 methylation and affects the malignant biological behaviors of glioma. Genomics.

[79] Pećina-Šlaus N, Hrašćan R (2024 Apr 19). Glioma stem cells-features for new therapy design. Cancers (Basel).

[80] Huang C, Qiu O, Mao C, Hu Z, Qu S (2022). An integrated analysis of C5AR2 related to malignant properties and immune infiltration of gliomas. Cancer Innov.

[81] Mao C, Huang C, Hu Z, Qu S (2022). Transcription factor CASZ1 increases an oncogenic transcriptional process in tumorigenesis and progression of glioma cells. MedComm.

[82] Qu S, Huang C, Zhu T, Wang K, Zhang H, Wang L (2023). OLFML3, as a potential predictor of prognosis and therapeutic target for glioma, is closely related to immune cell infiltration. VIEW.

[83] Qu S, Huang C, Hu Z The PODNL1/AKT/β-catenin signaling axis mediates glioma progression and sensitivity to temozolomide. Fundamental Research Online ahead of print.

